# Glutamine metabolism prognostic index predicts tumour microenvironment characteristics and therapeutic efficacy in ovarian cancer

**DOI:** 10.1111/jcmm.18198

**Published:** 2024-03-20

**Authors:** Lingling Gao, Zheng Wei, Feiquan Ying, Lin Huang, Jingni Zhang, Si Sun, Zehua Wang, Jing Cai, Yuan Zhang

**Affiliations:** ^1^ Department of Obstetrics and Gynecology, Union Hospital Tongji Medical College, Huazhong University of Science and Technology Wuhan China; ^2^ Department of Obstetrics and Gynecology Third Hospital of Shanxi Medical University, Shanxi Bethune Hospital, Shanxi Academy of Medical Sciences, Tongji Shanxi Hospital Taiyuan China

**Keywords:** metabolism reprogramming, molecular subtype, ovarian tumour, patient‐derived organoids (PDOs), single‐cell RNA transcriptome

## Abstract

Mounting evidence has highlighted the multifunctional characteristics of glutamine metabolism (GM) in cancer initiation, progression and therapeutic regimens. However, the overall role of GM in the tumour microenvironment (TME), clinical stratification and therapeutic efficacy in patients with ovarian cancer (OC) has not been fully elucidated. Here, three distinct GM clusters were identified and exhibited different prognostic values, biological functions and immune infiltration in TME. Subsequently, glutamine metabolism prognostic index (GMPI) was constructed as a new scoring model to quantify the GM subtypes and was verified as an independent predictor of OC. Patients with low‐GMPI exhibited favourable survival outcomes, lower enrichment of several oncogenic pathways, less immunosuppressive cell infiltration and better immunotherapy responses. Single‐cell sequencing analysis revealed a unique evolutionary trajectory of OC cells from high‐GMPI to low‐GMPI, and OC cells with different GMPI might communicate with distinct cell populations through ligand‐receptor interactions. Critically, the therapeutic efficacy of several drug candidates was validated based on patient‐derived organoids (PDOs). The proposed GMPI could serve as a reliable signature for predicting patient prognosis and contribute to optimising therapeutic strategies for OC.

## 1 INTRODUCTION

Ovarian cancer (OC) remained the most lethal malignancies among gynaecological tumours with a 5‐year survival rate less than 50% for all stages.^1^ The high mortality rate of OC is largely attributed to ambiguous and nonspecific symptoms, aggressive nature, chemoresistance and limited efficacy of immunotherapy.^2^, ^3^ In recent years, emerging evidence has strongly supported that as a crucial hallmark of cancer cells within the tumour microenvironment (TME), metabolic reprogramming provides the basis for tumour progression and therapeutic strategies, such as glycolysis, amino acid metabolism and fatty acid metabolism.^4^, ^5^ In this regard, targeting metabolism reprogramming in combination with chemotherapy or immunotherapy holds great promise in tumour treatment.^6^ Therefore, a deeper understanding of metabolic reprogramming in TME is necessary for the development of novel therapeutics for OC.

Glutamine metabolism (GM) has gained much attention as a therapeutic target for tumour cells.^7^ Glutamine, the most abundant nonessential/conditionally essential amino acid, plays an indispensable role in diverse and complex biological processes, including biomacromolecule synthesis (nucleotide, amino acid and lipid), replenishment of the tricarboxylic acid cycle, protein glycosylation, extracellular matrix production, epigenetic modifications, maintenance of intracellular redox homeostasis and glutathione.^4^, ^8^ Multiple malignancies have raised the requirement for glutamine to meet the elevated energy demand for survival.^9^ Glutamine dependence was associated with tumour aggressiveness and cisplatin resistance in OC.^10^ In addition, polyfactorial processes (glutamine transporters, glutaminase and aminotransferase) are involved in GM in various cancers.^8^ Consequently, targeting important molecules involved in GM, such as BPTES (GLS1/2 inhibitor), CB‐839 (GLS1 inhibitor), V9302 (SLC1A5 inhibitor), JHU‐083, holds great promise for cancer treatment.^11^, ^12^, ^13^, ^14^, ^15^

Accumulating evidence has shown that glutamine is an immunomodulatory metabolite involved in the activation, differentiation and antitumor immune response of immune cells.^16^, ^17^ For example, GM played a vital role in regulating the differentiation of T cells and B lymphocytes and the activation of macrophages.^16^ Although clinical trials of immunotherapy have demonstrated limited efficacy in ovarian cancer patients, combination therapy with GLS inhibitor 968 and anti‐PD‐L1 antibody boosted antitumor immunity and extended overall survival (OS) in OC patients.^18^ These researches suggest that treatment strategy of targeting GM in combination with immunotherapy shows broad prospects, indicating that deeper insight into GM may provide more prognostic biomarkers as well as the identification of novel potential targets for immunotherapy and chemotherapy in OC. However, the role of GM for patients' prognostic stratification and responsiveness to immunotherapy and chemotherapy in OC patients has not been fully elucidated.

In this study, employing multi‐omics analysis and integrative machine learning strategies, we comprehensively investigated the expression patterns of glutamine metabolism‐related genes (GMRGs) and identified three distinct GM clusters. Subsequently, a glutamine metabolism prognostic index (GMPI) consisting of 7 genes was established to quantify GM subtypes in OC. Furthermore, a comprehensive investigation was conducted to explore the association between both GM clusters, the GMPI and its prognostic implications, immune microenvironment and treatment response in various cohorts of ovarian cancers. Additionally, we explored evolutionary trajectory and intercellular communication based on GMPI at a single‐cell resolution. Patient‐derived organoids (PDOs) were utilised to assess the responsiveness of therapeutic agents. The GMPI model served as a powerful tool facilitating prognosis prediction and personalised treatment of OC patients, which contributed to improve patient outcome and advance individualised therapy in OC patients.

## 2 MATERIALS AND METHODS

### 2.1 Data acquisition and preprocessing

Table S1 shows the detailed information of the datasets enrolled in this study. Gene expression profiles and complete clinical characteristics of TCGA‐OV datasets were obtained from The Cancer Genome Atlas (TCGA) (https://portal.gdc.cancer.gov/), and gene expression data from 88 normal ovarian samples were retrieved from the GTEx database (https://xenabrowser.net/datapages/). The fragments per kilobase million (FPKM) were transformed into the transcripts per kilobase million (TPM) for subsequent analysis.^19^ Patients without OS information were excluded from this work.

Transcriptome expression datasets were obtained from the NCBI Gene Expression Omnibus (GEO) (https://www.ncbi.nlm.nih.gov/geo/). Three datasets (GSE14764, GSE23554 and GSE26712) were obtained from the GPL96 platform; five datasets (GSE26193, GSE63885, GSE18520, GSE19829 and GSE30161) from the GPL570 platform were merged and set as validation cohort 1 (GEO cohort‐1), and another four datasets from GPL6480 platform (GSE17260, GSE32062, GSE140082 and GSE49997) were used as validation cohort 2 (GEO cohort‐2). The sample size of each dataset was shown in Table S1. The expression values were normalised with the “RMA” method, and batch effects were corrected using the “Combat” algorithm with the “SVA” R package.^20^

### 2.2 Consensus molecular clustering of GMRGs

A total of 155 glutamine metabolism‐related genes (GMRGs) were extracted from the Molecular Signatures Database (MSigDB)^21^ (http://www.gseamsigdb.org). Patients from multiple cohorts were classified into distinct molecular subtypes based on GMRGs with survival significance with R package “ConsensusClusterPlus” through 100 repetitions and resampling of 80%.^22^

### 2.3 Construction of glutamine metabolism prognostic index (GMPI)

Differentially expressed genes (DEGs) among the three GM clusters were determined using R package “limma” with an adjusted *p* < 0.05 and |log2 FC| > 1. A total of 143 DEGs were further subjected to conduct univariate Cox regression analysis. To establish a scoring system regarding GM, the least absolute shrinkage and selection operator (LASSO) and Cox proportional hazards regression analysis were performed using “glmnet” R package with 1000 times.^23^ Forest plots were displayed with R package “forest plot”. Finally, seven genes were identified and included in the scoring system called “glutamine metabolism prognostic index (GMPI)”, which included KIAA0100, ANKRD27, OPA3, NUMBL, PDP1, SLC7A11 and TRMT112.

$$\text{GMPI}=\sum \left(\text{Coef}\;i^{*}\mathrm{Exp}\;i\right).$$

The Exp *i* represents the expression level of a specific gene, and Coef *i* represents the coefficient in multivariate Cox analysis. Kaplan–Meier (KM) survival analysis was performed to predict the prognosis of the different groups using “survminer” R package, and receiver operating characteristic (ROC) curves were generated to evaluate the predictive power of the biomarkers with “timeROC” R package.

### 2.4 Construction and validation of a predictive nomogram

Univariate and multivariate analyses were used to explore the independent prognostic biomarkers for OC. A nomogram, which incorporated GMPI and clinical parameters, was developed to quantify OS of OC patients using R package “rms”. The proportional hazards (PH) assumption was verified with the Schoenfeld test. Additionally, the 3‐ and 5‐year ROC values of the GMPI were compared with those of other published gene signatures for OC, including a prognosis‐related genes signature reported by Chen et al.^24^ (“Chen signature”), a prognostic model for platinum‐treated OC reported by Sabatier et al.^25^ (“Sabatier signature”), a lactate metabolism‐related signature reported by Xiang et al.^26^ (“Xiang signature”), a hematogenous and lymphatic metastasis signature reported by Yue et al.^27^ (“Yue signature”), and an omentum metastasis‐related prognostic signature reported by Zhang et al.^28^ (“Zhang signature”). Finally, the precision and accuracy of nomogram was evaluated with ROC curves, the Concordance index (C index) and restricted mean survival (RMS) time curves.

### 2.5 Gene set variation analysis (GSVA) and functional annotation

The differences in biological processes among different subtypes were determined by gene set variation analysis (GSVA)^29^ based on the hallmark gene set c2.cp.kegg.v7.4.symbols extracted from MSigDB database. Functional annotation was performed by the “ClusterProfiler” R package with a cut‐off value of adjusted *p* < 0.05. Gene Ontology (GO) and Kyoto Encyclopaedia of Genes and Genomes (KEGG) analyses were performed, and the term with adjusted *p* < 0.05 was regarded as statistical significance.

### 2.6 Estimation of immune cell infiltration in TME of OC

The single‐sample gene‐set enrichment analysis (ssGSEA) was employed to evaluate the relative abundance of each type immune cell infiltration in the TME of OC with the “GSVA” R package.^30^ Estimation of Stromal and Immune Cells in Malignant Tumours using Expression Data (ESTIMATE) algorithm was utilised to estimate the stromal score, immune score and ESTIMATE score, predicting the level of infiltrating immune cells and tumour purity in OC.^31^ We also utilized the XCELL, QUANTISEQ, TIMER, MCPCOUNTER, EPIC, CIBERSORT‐ABS and CIBERSORT to quantify the immunogenomic landscape of immune infiltration in OC. In addition, the correlations between genes associated with immune checkpoints, chemokines, interleukins, as well as epithelial‐mesenchymal transition (EMT) and different GMPI groups were further explored.

### 2.7 Estimation of drug susceptibility and immunotherapy response

The expression data of human cancer cell lines (CCLs) were collected from the Broad Institute Cancer Cell Line Encyclopaedia (CCLE) project (https://portals.broadinstitute.org/ccle/).^32^ Drug sensitivity data of CCLs was calculated from the Cancer Therapeutics Response Portal (CTRP) (https://portals.broadinstitute.org/ctrp) (481 compounds against 860 CCLs) and PRISM repurposing dataset (https://depmap.org/portal/prism/) (1448 compounds against 499 CCLs). Expression data in CCLE were employed for subsequent CTRP and PRISM analysis. K‐nearest neighbour (k‐NN) algorithm was subjected to calculate the missing AUC values. The area under the dose–response curve (AUC) was utilised to evaluate drug sensitivity, and a lower AUC value indicated higher sensitivity to drugs.

The Tumour Immune Dysfunction and Exclusion (TIDE) algorithm (http://tide.dfci.harvard.edu/) was used to explore immunotherapy response and immune evasion based on the TCGA‐OC cohort.^33^ Immunophenoscore (IPS) was employed to evaluate the relationship of GMPI with patients' response to anti‐PD‐1 and anti‐CTLA4 therapy.^30^ IPS scores for TCGA‐OC patients were extracted from The Cancer Immunome Atlas (TCIA) database (https://tcia.at/home). Moreover, the immunotherapeutic cohort (IMvigor210 dataset), which included advanced urothelial cancer patients treated with anti‐PD‐L1 antibody (atezolizumab), was also employed to evaluate immunotherapy efficacy in patients with different GMPI groups.^34^

### 2.8 Single‐cell RNA sequencing (**scRNA‐seq**) analysis of ovarian cancer

ScRNA‐seq dataset from GSE181955 was selected for data reprocessing and cell classification with the “Seurat” R package.^35^ “SCTransform” was used to normalise data and highly variable genes (HVGs) were further identified with the “FindVariableFeatures” function. Top 3000 HVGs were subsequently subjected to principal component analysis (PCA) for dimension reduction. The cell clusters were visualised using the “uniform manifold approximation and projection (UMAP)” method. Cells were annotated according to the marker genes of different cell types. The Wilcox algorithm was used to analyse DEGs with statistical significance in each manually modified cluster using “FindAllMarkers” function (adjusted *p* < 0.05). Intercellular communication among various cell types was evaluated by the “CellChat” package.^36^ Pseudotime trajectory analysis of single cells were conducted with “Monocle 2” package. The “UCell” package was introduced to evaluate expression of seven GMPI‐related genes at the single‐cell level via “AddModuleScore_UCell” algorithm.

### 2.9 Quantitative real‐time PCR (qRT‐PCR)

Tissue RNA was isolated by using TRIzol reagent (TaKaRa, Japan), and reverse transcription was conducted with a HiScript®III RT SuperMix Kit (Vazyme, China) according to the manufacturer's procedures. PCR amplification was performed using AceQ qPCR SYBR Green Master Mix (Vazyme, China). The ΔCt values of seven genes (*KIAA0100*, *ANKRD27*, *OPA3*, *NUMBL*, *PDP1*, *SLC7A11* and *TRMT112*) in each sample were calculated and included in the scoring system: GMPI = ∑ (coef i*Exp i). *GAPDH* served as the reference gene. The primer sequence information is shown in Table S2.

### 2.10 Establishment of patient‐derived organoids (PDOs)

Biological samples obtained from OC patients were collected according to the principles of the Declaration of Helsinki and approved by The Ethics Committee of Tongji Medical College, Huazhong University of Science and Technology. Fresh OC specimens were obtained by surgery, cut into small pieces (<1 mm^3^) and digested into single‐cell suspensions. Then, the suspension was mixed with Matrigel and plated into a 96‐well plate, and cultured in medium composed of Advanced DMEM/F12 supplemented with 1% penicillin streptomycin, 1 × Glutamax (Gibco, 35,050,061), 1% HEPES (Stemcell, 07200), 1 × B27 (Stemcell, 05731), 100 ng/mL Noggin (Peprotech, 120‐10C), 50 ng/mL EGF (Peprotech, AF‐100‐15), 100 ng/mL R‐spondin 1 (Peprotech, 120‐38), 10 ng/mL FGF‐10 (Peprotech, 10,026), 10 ng/mL FGF2 (Peprotech, 100‐18B), 0.02 μg/mL Wnt3a (R&D systems, 5036‐WN), 0.05 μg/mL human NRG1 (R&D systems, 5898‐NR), 10 mM nicotinamide (Sigma Aldrich, N0636), 1.25 mM N‐acetylcysteine (Sigma Aldrich, A9165), 10 nM 17‐β‐Estradiol (R&D systems, 2824), 10 μM SB202190 (Sigma Aldrich, S7076), 500 nM A83‐01 (Sigma Aldrich, SML0788) and Y‐27632 dihydrochloride (Stemcell, 72,302). For drug treatment, the working concentrations of these drugs were as follows: 15 μM cisplatin (Selleck, S1166), 1 μM paclitaxel (Selleck, S1150), 100 nM panobinostat (Aladdin, P125167), 1 μM gemcitabine (Aladdin, G127944) and 5 μM prexasertib (Selleck, S6385). The control group was treated with the same volume of PBS or DMSO. After 4 days of drug treatment, the growth and morphology of organoids were observed and photographed under a microscope.

### 2.11 CellTiter‐Glo‐3D assay

The cell viability of the organoids was detected by a CellTiter‐Glo 3D assay (Promega, G9618) according to the manufacturer's instructions. Briefly, the culture medium of organoids was discarded, and a detection reagent was added. The cells were then incubated for 10 min at room temperature, and luminescence was measured at 560 nm by a microplate reader.

### 2.12 Statistical analysis

R software (version 4.1.1) was used for all the statistical analyses. The statistical significance of difference between two groups was calculated by Student's *t* test and Mann–Whitney *U* test as appropriate. Comparisons among more than two groups was estimated by one‐way analysis of variance (ANOVA) and the Kruskal–Wallis test. Kaplan–Meier analysis and log‐rank test were employed for survival analysis. Univariate and multivariate Cox regression analyses were utilised to identify independent prognostic indicators. Spearman analysis was conducted to calculate the correlation coefficient for non‐normally distributed continuous variables. A two‐side *p* value < 0.05 was considered as statistical significance.

## 3 RESULTS

### 3.1 Landscape of genetic characteristics and transcriptional patterns of glutamine metabolism‐related genes (GMRGs) in OC

**FIGURE 1:**
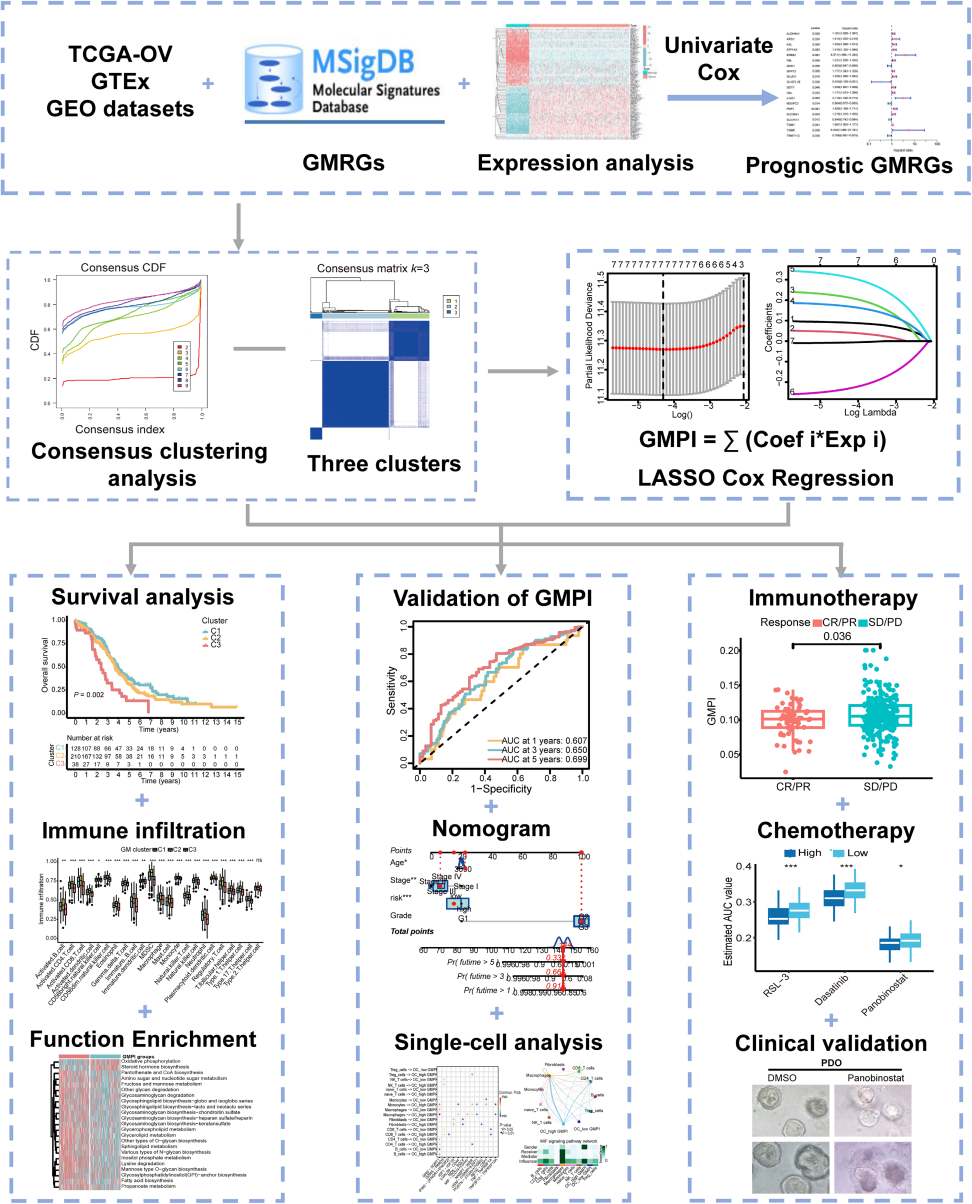
The flow chart of this study.

The flow chart detailing this study is shown in Figure 1. A total of 135 GMRGs were ultimately identified and obtained from MSigDB and published articles. We first evaluated the mRNA expression of these GMRGs between the TCGA‐OV cohort (381 OC tissues) and the GTEx cohort (88 normal tissues). Seventy‐seven of these genes were differentially expressed (all *p* < 0.05; Figure S1A), and 20GMRGs were significantly associated with the OS of patients (all *p* < 0.05) and were selected for further analysis (Figure S1B and Table S3). Among them, the expression levels of 10 genes (*ALDH4A1*, *GLUD1*, *GLYATL1B*, *GOT1*, *PDP1*, *SLC38A1*, *SLC7A11*, *TGM1*, *TGM6* and *TRMT112*) were upregulated (all *p* < 0.05), and the expression levels eight genes (*ARG1*, *ASL*, *EPB42*, *FBL*, *GAD1*, *GFPT2*, *KYAT1* and *NDUFC2*) were downregulated (all *p* < 0.05) (Figure S1C). The comprehensive landscape of 20 GMRGs interactions was shown in Figure S1D. There were positive correlations between ATP1A3 and GOT1 and between NDUFC2 and HAL. We further analysed the CNV amplification and deletion of 20 GMRGs, *NDUFC2*, *PDP1*, *GFPT2*, *FBL*, *GAD1*, *TGM1*, *TGM6*, *SLC38A1*, *TRMT112*, *ASL* and *SLC7A11* showed widespread CNV amplification, and *ATP1A3*, *ALDH4A1*, *KYAT1*, *GLUD1*, *ARG1*, *HAL*, *GOT1* and *EPB42* showed widespread CNV deletions (Figure S1E). The locations of CNV alterations in these GMRGs on the chromosomes were shown in Figure S1F. These results indicated high heterogeneity of genomic and transcriptomic alteration in GMRGs of OC patients, suggesting that GM might play a crucial role in OC development and progression.

### 3.2 Identification of GM clusters in OC

**FIGURE 2:**
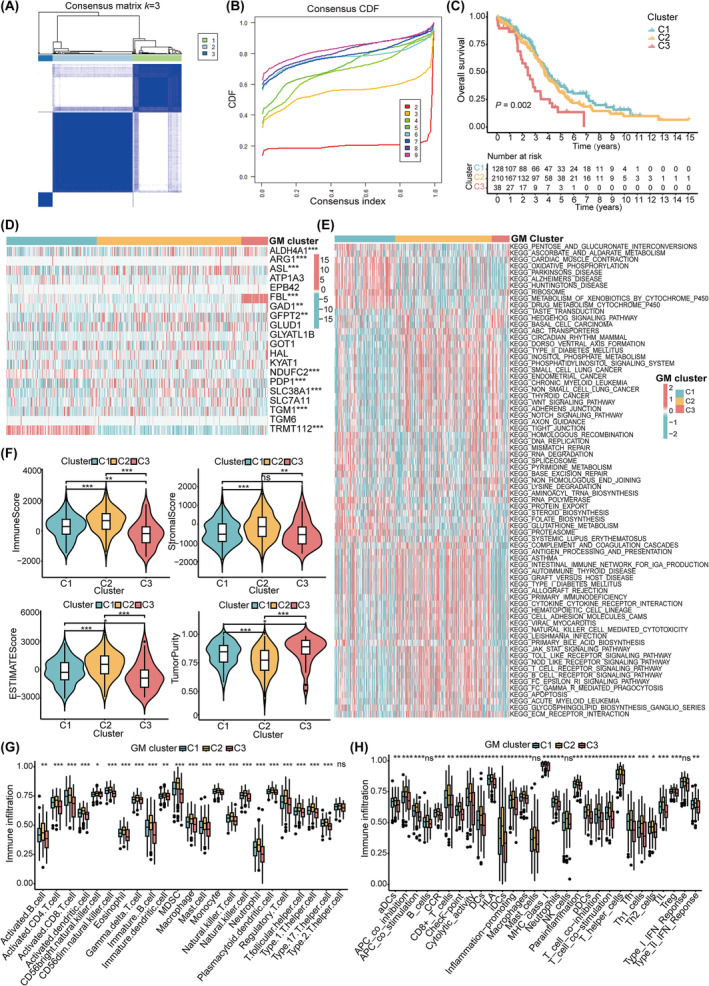
Biological characteristics and immune infiltration heterogeneity among three clusters based on 20 GMRGs with prognostic values. (A) Consensus clustering matrix of sample under *k* = 3. (B) Cumulative distribution curve with the number of subtypes *k* = 2–9. (C) Survival analysis of three clusters based on TCGA‐OV cohort with Kaplan–Meier curves. (D) Differences in mRNA expression levels of 20 GMRGs among three clusters shown by heatmap. (E) Pathway enrichment analysis among three clusters with GSVA shown by heatmap. (F) Differences of immune score, stromal score, ESTIMATE score and tumour purity among 3 GM clusters with violin plot. (G) Immune cells infiltration in three clusters analysed with ESTIMATE algorithm. (H) Potential immune function in three clusters analysed with ESTIMATE algorithm. *p < 0.05; **p < 0.01; ***p < 0.001.

To further explore the involvement of GMRGs in OC, we performed consensus clustering analysis to stratify the patients from TCGA‐OV database based on the expression of 20 GMRGs with prognostic values. Accordingly, *k* = 3 was identified as an optimal selection for classifying the entire sample into three clusters, which were defined as GM‐C1 (*n* = 128 samples), GM‐C2 (*n* = 212 samples) and GM‐C3 (*n* = 38 samples) (Figure 2A,B). Survival analysis indicated that GM‐C1 was associated with a prominent survival advantage, whereas GM‐C3 displayed a worse survival prognosis. The trend of survival curve showed that the OS time of GM‐C2 was between C1 and C3 (*p* = 0.002) (Figure 2C). The expression levels of NDUFC2 and TRMT112 were upregulated in GM‐C1, and the expression levels of ALDH4A1, FBL and SLC38A1 were upregulated in GM‐C3 (Figure 2D). In addition, we performed identical analyses with the GPL570 cohort (Figure S2A,C) and GPL96 cohort (Figure S2D–F), and similar results were observed (all *p* < 0.05).

We further explored the biological functions characterising the three distinct clusters. GSVA enrichment analysis revealed that biological processes such as ribosomes, oxidative phosphorylation and cardiac muscle contraction were highly activated in GM‐C1. Pathways associated with immune regulation were significantly enriched in GM‐C2, these pathways included Fc epsilon RI signalling pathway, Fc gamma R mediated phagocytosis, the Toll‐like receptor signalling pathway, B‐cell receptor signalling pathway and T‐cell receptor signalling pathway. GM‐C3 exhibited enrichment in pathways related to nonhomologous end joining, the hedgehog signalling pathway and the notch signalling pathway (Figure 2E). Through ESTIMATE algorithm, we confirmed that GM‐C3 had the lowest immune score, stromal score, ESTIMATE score and the highest tumour purity (all *p* < 0.05; Figure 2F). Additionally, ssGSEA analysis indicated significant differences in the infiltration of most immune cells among different clusters, GM‐C3 exhibited the lowest infiltration levels of various immune cells, including activated B cells, activated CD4^+^ T cells, activated CD8^+^ T cells and natural killer T cells (all *p* < 0.05; Figure 2G). Moreover, various DCs (aDCs, DCs, iDCs and pDCs), T cells and NK cells, APC co‐stimulation, T cell co‐stimulation, HLA and type II IFN responses were significantly enriched in GM‐C2, which suggested highly active antigen presentation and antitumor immunity in GM‐C2 (all *p* < 0.05; Figure 2H).

### 3.3 Construction of the glutamine metabolism prognostic index (GMPI)

**FIGURE 3:**
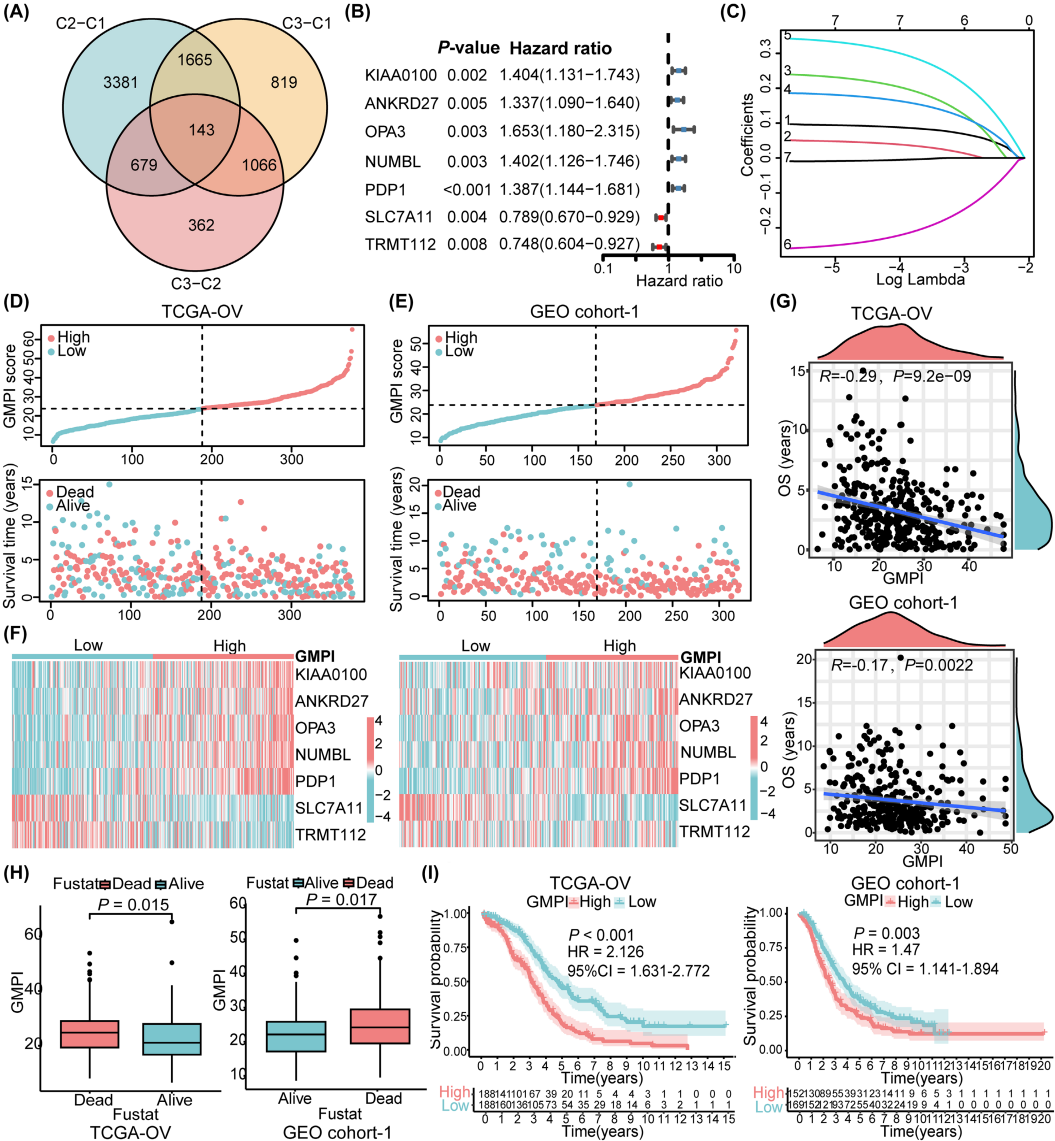
Construction and correlation analysis of GMPI in OC. (A) Intersection of DEGs among 3 GM clusters shown by Venn diagram. (B) Prognosis‐related DEGs via univariate Cox regression analysis with *p* < 0.01. (C) Screening diagnostic markers with LASSO logistic regression model with a filtering threshold of *p* < 0.05. (D, E) GMPI distribution and expression profiles of seven prognostic genes in low‐ and high‐ GMPI groups from patients in TCGA‐OV cohort and GEO cohort‐1. (F) Different expression levels of seven robust genes in low‐ and high‐ GMPI groups shown by heatmap. (G) Correlation between GMPI and OS of OC patients from TCGA‐OV cohort and GEO cohort‐1. (H) Relationship between GMPI level and mortality rate of patients from TCGA‐OV cohort and GEO cohort‐1. (I) OS of patients in low‐ and high‐ GMPI groups from TCGA‐OV cohort and GEO cohort‐1 shown by Kaplan–Meier survival curves. *p < 0.05; **p < 0.01; ***p < 0.001.

To investigate the potential biomolecular characteristics underlying the different GM subtypes, we identified 143 DEGs among the 3 GM clusters and illustrated in a Venn diagram (Figure 3A). GO enrichment analysis indicated that most significant terms were related to the biological process of histone modification and mRNA processing, cellular component of histone deacetylase complex, and molecular function of DNA‐binding transcription activator activity (Figure S3A), KEGG analysis revealed that these DEGs were mostly involved in the pathway of herpes simplex virus 1 infection and PD‐L1 expression and PD‐1 checkpoint (Figure S3B).

Univariate Cox regression analysis was utilized to screen out 49 DEGs with significant prognostic value in OC patients (all *p* < 0.05), these DEGs were defined as glutamine metabolism related signature genes (GMRSGs; Table S4). Subsequently, we conducted LASSO Cox regression analysis with the 49 GMRSGs and 20 GMRGs with prognostic value (Figure S4A), and obtained 7 robust genes (KIAA0100, ANKRD27, OPA3, NUMBL, PDP1, SLC7A11 and TRMT112) with statistical significance (all *p* < 0.01; Figure 3B,C, Figure S4B–H). Finally, the index model was developed as follows: GMPI = 0.090* KIAA0100 + 0.043* ANKRD27 + 0.215* OPA3 + 0.171* NUMBL + 0.314* PDP1–0.233*SLC7A11–0.007* TRMT112. According to the median value of the GMPI, patients from TCGA‐OV cohort (training dataset) and GEO cohort‐1 (validation dataset) were divided into low‐ and high‐ GMPI groups. The distributions of patients in the live and dead groups were shown in Figure 3D,E. As the GMPI increased, the mortality of patients increased (Figure 3D,E). The heatmap showed different expression levels of seven robust genes in high‐ and low‐ GMPI groups, and expression levels of KIAA0100, ANKRD27, OPA3, NUMB and PDP1 were upregulated in high‐GMPI group, while SLC7A11 and TRMT112 were downregulated in low‐GMPI group (all *p* < 0.05) (Figure 3F). The GMPI was negatively correlated with the OS of OC patients from TCGA‐OV cohort (*R* = −0.29, *p* = 9.2e‐09) and GEO cohort‐1 (*R* = −0.17, *p* = 0.0022), patients with worse survival outcomes displayed high GMPI risk scores (all *p* < 0.05) (Figure 3G,H). Afterward, Kaplan–Meier analysis of the training dataset showed the low‐GMPI group had a significant improvement in survival compared with the high – GMPI group (HR = 2.126, 95%CI = 1.631–2.772, *p* < 0.001), as did the GEO cohort‐1 (HR = 1.47, 95%CI = 1.141–1.894, *p =* 0.003), respectively (Figure 3I).

### 3.4 Evaluation and Validation of the GMPI

**FIGURE 4:**
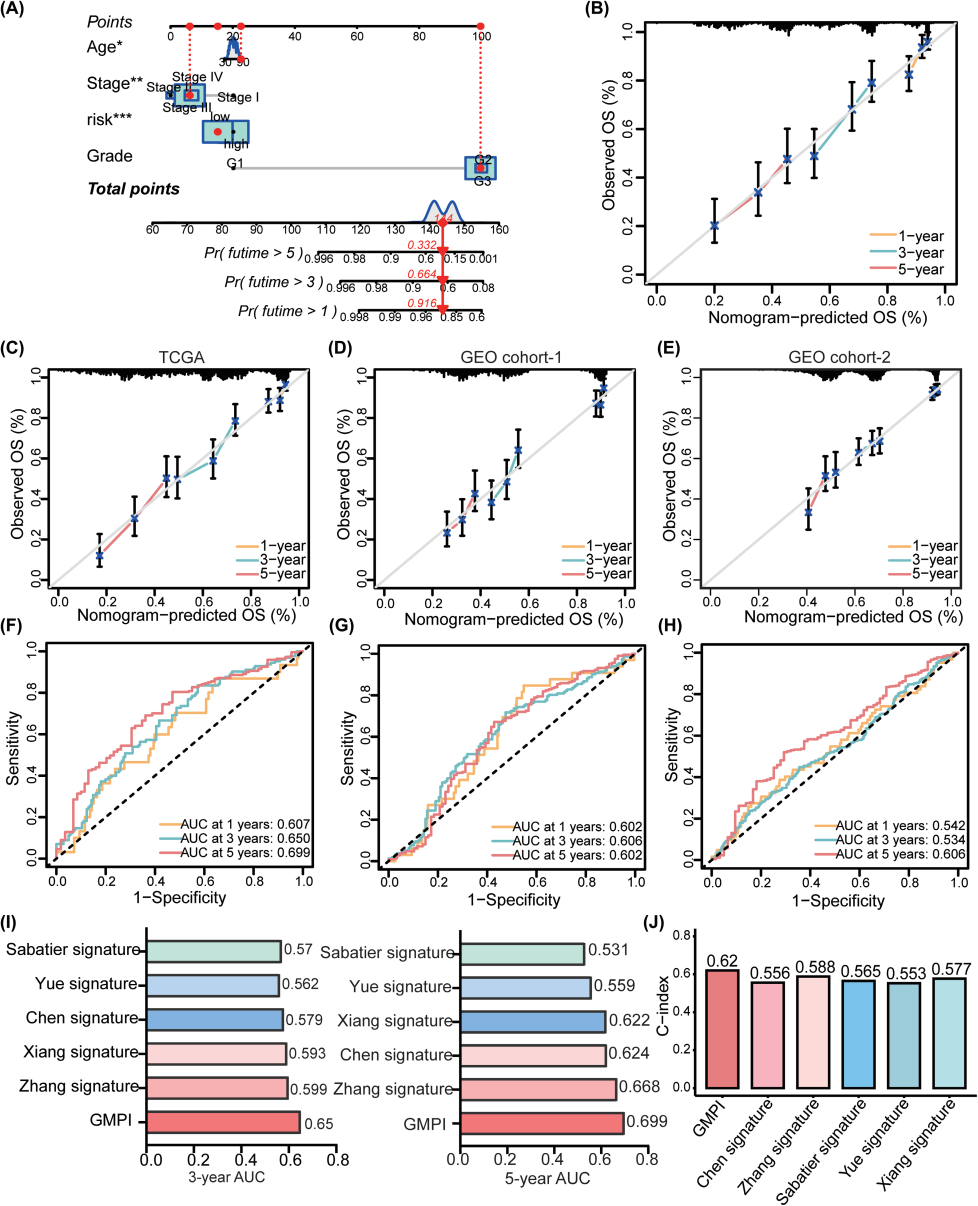
Validation and performance evaluation of GMPI in OC. (A) Construction of a nomogram for predicting the probability of 1‐, 3‐ and 5‐year OS in OC based on GMPI and clinical characteristics. (B) The probability and consistency of 1‐, 3‐ and 5‐year OS predicted by nomogram with calibration curves. (C‐E) The probability and consistency of 1‐, 3‐ and 5‐year OS with calibration curves based on TCGA‐OV cohort, GEO cohort‐1 and GEO cohort‐2. (F–H) The prediction performance of GMPI of 1‐, 3‐ and 5‐year OS shown by ROC curves based on TCGA‐OV cohort, GEO cohort‐1 and GEO cohort‐2. (I) Comparison of AUC in 3‐year and 5‐year OS for GMPI and other five published signatures. (J) C‐index of six prognostic models constructed for OC. *p < 0.05; **p < 0.01; ***p < 0.001.

Univariate and multivariate regression analyses showed that GMPI was an independent prognostic factor in OC (Figure S5A). Based on the results of multivariate analysis, a nomogram was constructed based on GMPI, age, FIGO stage and tumour grade (Figure 4A). Calibration curves showing the predicted and actual 1‐, 3‐ and 5‐year survival rates indicated the ideal consistency of the nomogram (Figure 4B–E). Furthermore, the accuracy of the GMPI was tested by time‐dependent ROC analysis. For the TCGA‐OV training cohort, the area under the ROC curve (AUC) was 0.607, 0.650 and 0.699 for 1‐year, 3‐year and 5‐year survival, respectively (Figure 4F). Moreover, ROC curve analysis of the validation dataset GEO cohort‐1 and GEO cohort‐2 revealed that GMPI had predictive performance (GEO cohort‐1: AUC = 0.0.602 for 1‐year survival, 0.606 for 3‐year survival and 0.602 for 5‐year survival; GEO cohort‐2: AUC = 0.542 for 1‐year survival, 0.534 for 3‐year survival and 0.606 for 5‐year survival; Figure 4G,H), suggesting that GMPI could better predict the survival status of OC patients. Compared with several other published signatures and popular biomarkers, GMPI had the highest AUC for either 3‐year (0.65) or 5‐year (0.699) survival (Figure 4I), and the C‐index of GMPI was highest with 0.62 than other types of models (Figure 4J). Besides, we also plotted the ROC curve and KM survival curves of each model (Figure S5B–E). We further evaluated the predictive effect of prognostic index at different time points. As a result, compared with the other models, GMPI had the best performance at a duration greater than 5 years, which indicated that GMPI possessed favourable predictive value for both patients with a survival time greater than 5 years and patients with a survival time less than 5 years (Figure S5F). The above results indicate that GMPI is a highly reliable signature and maintains good prediction performance.

### 3.5 Molecular function and immune infiltration heterogeneity of GMPI

**FIGURE 5:**
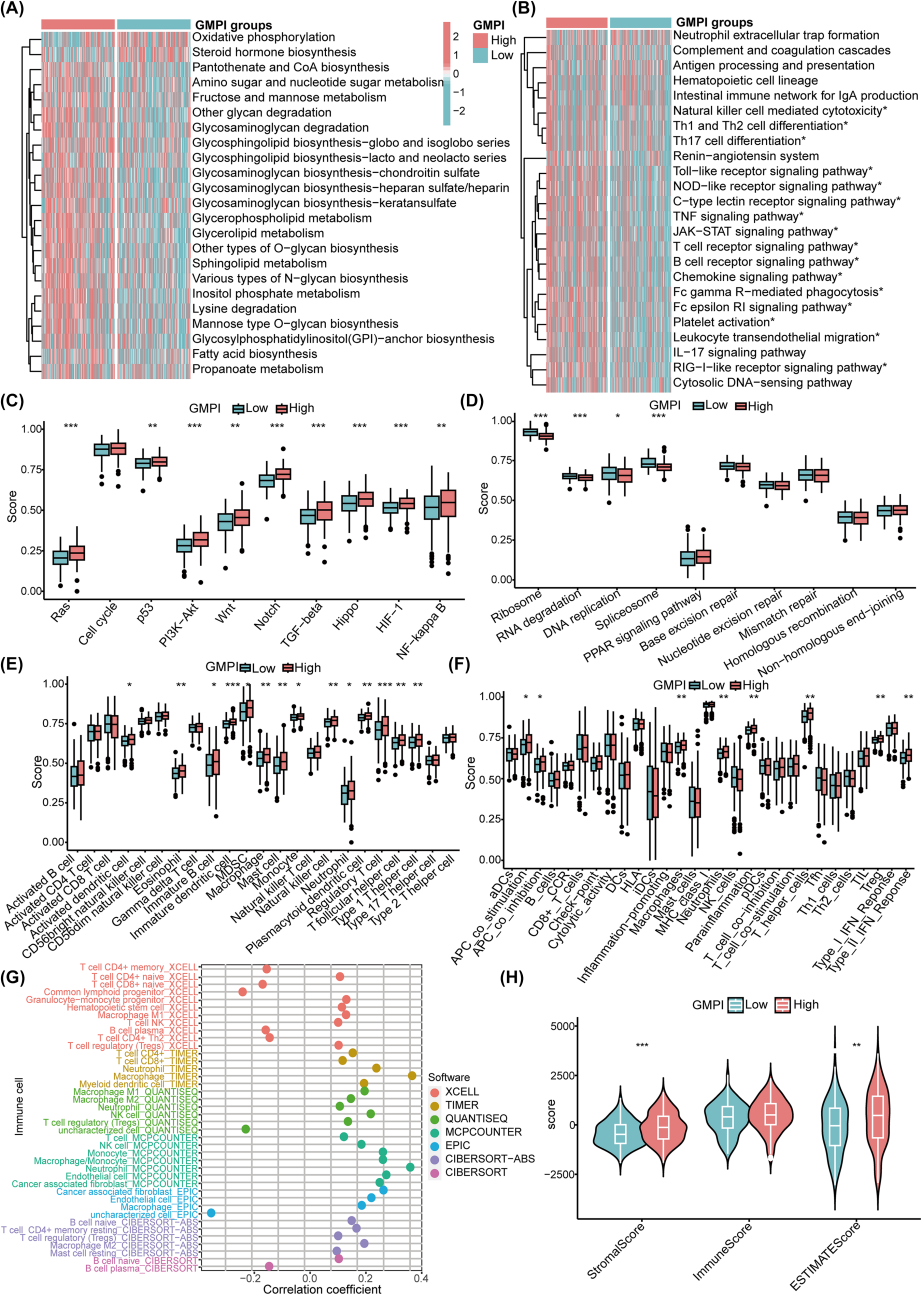
Characterization of hallmark pathways and infiltration characteristics of TME cells in low‐ and high‐GMPI groups. (A) Differences of metabolism‐related pathways between low‐ and high‐GMPI groups. (B) Differences of immune‐related pathways between low‐ and high‐GMPI groups. (C) Differences of cancer‐related pathways between low‐ and high‐GMPI groups. (D) Differences of genetic processes‐related pathways between low‐ and high‐GMPI groups. (E) Differences of tumour‐infiltrating immune cells between low‐ and high‐GMPI groups. (F) Different immune function between low‐ and high‐GMPI groups. (G) Correlation between GMPI and tumour‐infiltrating immune cells evaluated by TIMER, CIBERSORT, CIBERSORT−ABS, QUANTISEQ, MCP_counter, XCELL and EPIC algorithms. (H) Immune score, stromal score and ESTIMATE score in different GMPI groups analysed by Estimate algorithm. *p < 0.05; **p < 0.01; ***p < 0.001.

To explore the functional status associated with GMPI, we performed GSVA for all KEGG signalling pathways in the low‐ and high‐GMPI groups. A total of 23 metabolic pathways showed significant difference between low‐ and high‐GMPI groups (all *p* < 0.05; Figure 5A). Oxidative phosphorylation and steroid hormone biosynthesis were highly activated in low‐GMPI group, and glycosaminoglycan biosynthesis, glycosphingolipid biosynthesis, inositol phosphate metabolism, lysine degradation and fatty acid biosynthesis were upregulated in high‐GMPI group (all *p* < 0.05; Figure 5A). Furthermore, 16 immune‐related pathways, including B cell receptor signalling pathway, T cell receptor signalling pathway, Toll‐like receptor signalling pathway and TNF signalling pathway, were significantly enriched in the high‐GMPI group (all *p* < 0.05; Figure 5B). In addition, we analysed the expression of 10 cancer‐related signalling pathways, most of which were highly expressed in high‐GMPI group; these pathways included Notch, PI3K‐AKT, TGF‐beta and RAS signalling pathway (all *p* < 0.05; Figure 5C). Whereas genes associated with genetic information processing pathways (ribosome, RNA degradation, DNA replication and spliceosome) were significantly upregulated in the low‐GMPI group (all *p* < 0.05; Figure 5D).

We further utilised ssGSEA algorithm to estimate the proportions of 23 immune cells and immune‐related functions between low‐ and high‐GMPI groups and revealed that the infiltration levels of TME cells were significantly upregulated in the high‐GMPI group, including dendritic cells (DCs) (activated DCs, immature DCs, plasmacytoid DCs), NK T cells, T cells (regulatory T cells, T follicular helper cells, Type 1 T helper cells), eosinophils, immature B cells, MDSC, macrophages, mast cells and monocytes (all *p* < 0.05; Figure 5E). The immune functional status, such as APC co‐inhibition, APC co‐stimulation, macrophages, T helper cells, Treg and type II IFN response, was highly enriched in high‐GMPI group (all *p* < 0.05; Figure 5F). We also showed that GMPI was significantly associated with B cell naïve_CIBERSORT, B cell plasma_CIBERSORT, M2 macrophage_EPIC, cancer associated fibroblast_EPIC, endothelial cell_EPIC and T cell regulatory (Tregs)_XCELL et al (all *p* < 0.05) (Figure 5G and Table S5). We further confirmed that high‐GMPI group possessed high stromal ESTIMATE scores, respectively (all *p* < 0.05; Figure 5H).

### 3.6 Prediction of immunotherapy efficacy in patients with different GMPI

**FIGURE 6:**
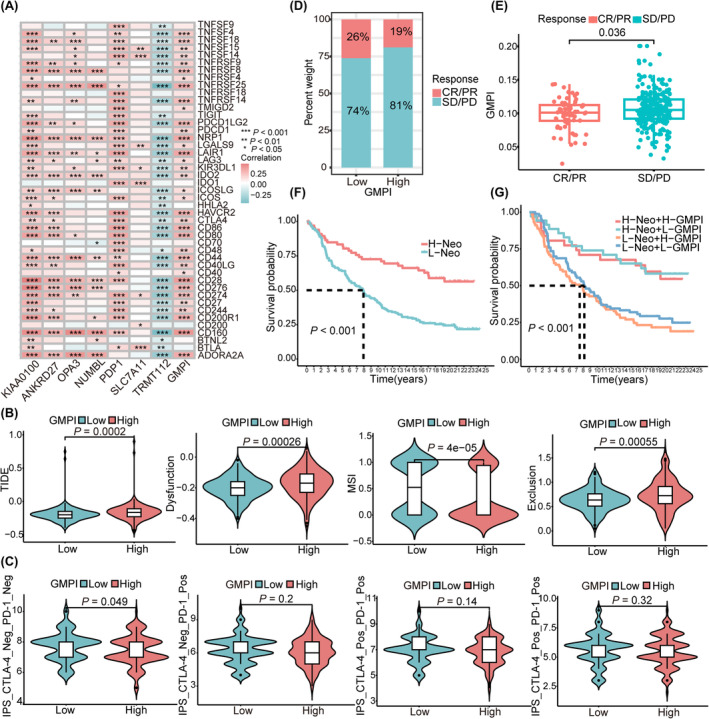
The relationship between GMPI and immunotherapy response. (A) Correlation analysis of immune checkpoints with GMPI‐related seven gene signatures. (B) The relationship of TIDE score and GMPI. (C) Distribution of IPS score in different GMPI groups based on TCGA‐OV datasets. Neg represents no response to treatment, Pos represents response to treatment. (D) Different anti‐PD‐L1 responsiveness between low‐ and high‐GMPI groups based on IMvigor210 database. CR: complete response, PR: partial response, SD: stable disease, PD: progressive disease. (E) The GMPI levels in different immunotherapy responsiveness based on IMvigor210 database. (F) Survival analysis of patients in low and high neoantigen burden groups in IMvigor210 database by Kaplan–Meier curve. H‐Neo: high neoantigen burden, L‐Neo: low neoantigen burden. (G) Survival analysis of patients with anti‐PD‐L1 immunotherapy stratified by both GMPI and neoantigen burden with Kaplan–Meier curves. *p < 0.05; **p < 0.01; ***p < 0.001.

Therapeutic response to immune checkpoint inhibitors has emerged as a promising strategy for cancer treatment. To investigate the potential role of GMPI for predicting immunotherapy responsiveness, we first analysed the correlation between seven GMPI‐related gene signatures and common immune checkpoints, and found that GMPI and genes (KIAA0100, ANKRD27, OPA3, NUMBL and PDP1) were positively correlated with most of the presented immune checkpoints (all *p* < 0.05; Figure 6A). SLC7A11 was correlated with the expression of CD274, IDO1, CD200, BTLA, LGALS9 and TNFSF14/15, whereas TRMT112 negatively correlated with the presented immune checkpoints (all *p* < 0.05; Figure 6A). The same trend was also found for the correlation between GMPI and the expression of chemokines, interleukins, MHCs and regulators related to the TGF‐β/EMT pathway (Figure S6A–D). Subsequently, with TIDE algorithm, we found that patients in high‐GMPI group showed higher TIDE score, dysfunction score and exclusion score, suggesting enhanced immune escape and poor response to immunotherapy (anti‐PD‐1 and anti‐CTLA‐4; all *p* < 0.05; Figure 6B). We further explored the correlation between GMPI and IPS score from TCIA database and found that the IPS_CTLA‐4_Neg/PD‐1_Neg score was significantly higher in low‐GMPI group (*p* < 0.05; Figure 6C). To further validate the predictive ability of the GMPI, the IMvigor210 cohort was employed to evaluate patients' response to immunotherapy. A higher proportion of patients with complete response/partial response (CR/PR) was observed in low‐GMPI group, contrasted by a higher number of patients with stable disease/progressive disease (SD/PD) in the high‐GMPI group (*p* < 0.05; Figure 6D). Furthermore, patients who achieved CR/PR presented significantly lower GMPI than those with SD/PD (*p* < 0.05; Figure 6E). Patients with a high neoantigen burden showed favourable prognosis compared with those with a low neoantigen burden (*p* < 0.001; Figure 6F), and patients with the combination of high neoantigen burden and low‐GMPI presented survival advantage (*p* < 0.001, Figure 6G). Collectively, these results provide evidence to support the ability of GMPI to predict immunotherapy efficacy, suggesting that patients in the low‐GMPI group could potentially benefit from immunotherapy.

### 3.7 The role of GMPI analysed at single‐cell resolution

**FIGURE 7:**
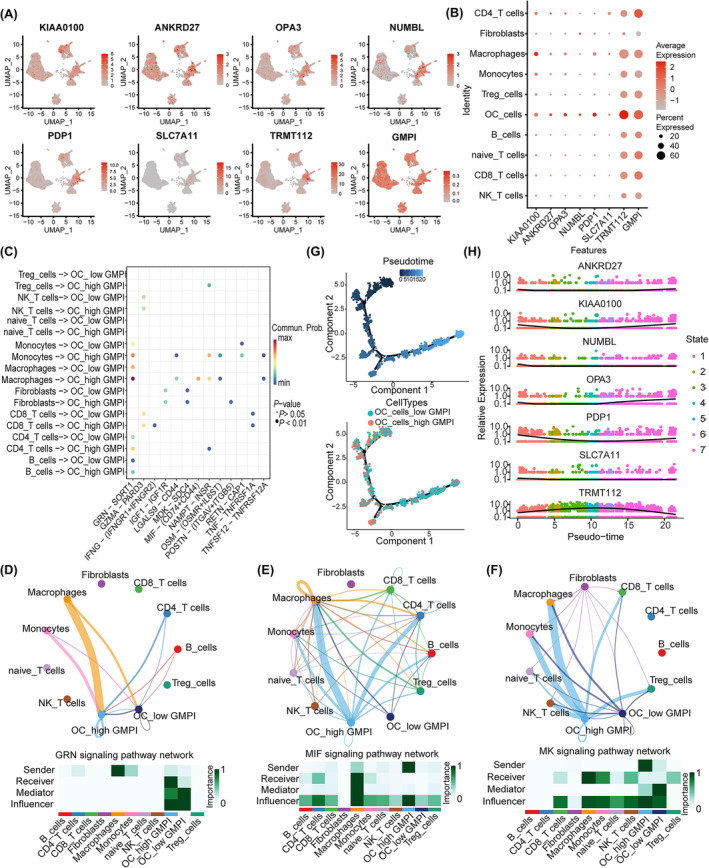
Intratumoral crosstalk and evolutionary trajectory of different GMPI groups in OC. (A, B) Distribution and expression levels of seven genes and GMPI with single‐cell RNA‐seq analysis. (C) Overview of representative ligand‐receptor interactions between distinct cell types and malignant cells in low‐ and high‐GMPI groups with dot plot. (D‐E) The networks of GRN signalling pathway (D), MIF signalling pathway (E), and MK signalling pathway (F) shown by circle plots, the function of different cell types in the different pathway networks shown by heatmaps. (G) Pseudotime analysis of trajectory differentiation from OC cells with high‐GMPI group into low‐GMPI inferred by monocle 2. (H) Relative expression of GMPI‐related seven genes in the differentiation process of OC cells coloured by cell states.

Recently, single‐cell sequencing has provided novel insight into intratumor heterogeneity in OC, and we further analysed the function of GMPI and its' interactions with various cells in TME at the single‐cell level. Fourteen clusters were identified with UMAP after cell quality (Figure S7A). The distributions of samples and cell types are shown in Figure S7B,C. These cells were subsequently clustered and annotated according to specific expression of marker genes, and eleven cell types were ultimately identified (Figure S7D). The proportions of different cell subtypes in each sample or in different tumour types were shown in Figure S7E. Increased expression levels of KIAA0100, ANKRD27, OPA3, NUMBL, PDP1 and TRMT112 were observed in OC cells (Figure 7A,B). We further performed intercellular interaction analysis based on ligand‐receptor pairs to explore the communication dynamics between different subtypes and malignant cells in low‐ and high‐GMPI groups (Figure S8A,B). Our findings indicated that cells with different GMPI were involved in interactions with various cell subtypes (Figure 7C). As illustrated, high‐GMPI OC cells could receive signals from various immune cells, such as macrophages, monocytes, CD4_T cells, Tregs cells and B cells, through GRN signalling pathway, MIF signalling pathway, MK signalling pathway and GALECTIN signalling pathway (Figure 7D–F and Figure S8C). A trajectory analysis was applied to investigate the role of GMPI in the dynamic expression pattern. The pseudotime results showed that high‐GMPI OC cells ultimately transited to low‐GMPI OC cells after experiencing seven diverging cell fates (Figure 7G and Figure S8D). Along the trajectory, the expression levels of GMPI‐related genes, such as KIAA01000, NUMBL, OPA3 and PDP1, were gradually decreased first and then increased during the transition, but that of TRMT112 exhibited the opposite trend (Figure 7H and Figure S8E,F). As a result, GMPI provides guidance for diverse cell communications and has an impact on tumour cell survival.

### 3.8 Correlation between the GMPI and drug sensitivity and validation with patient‐derived organoids (PDOs)

**FIGURE 8:**
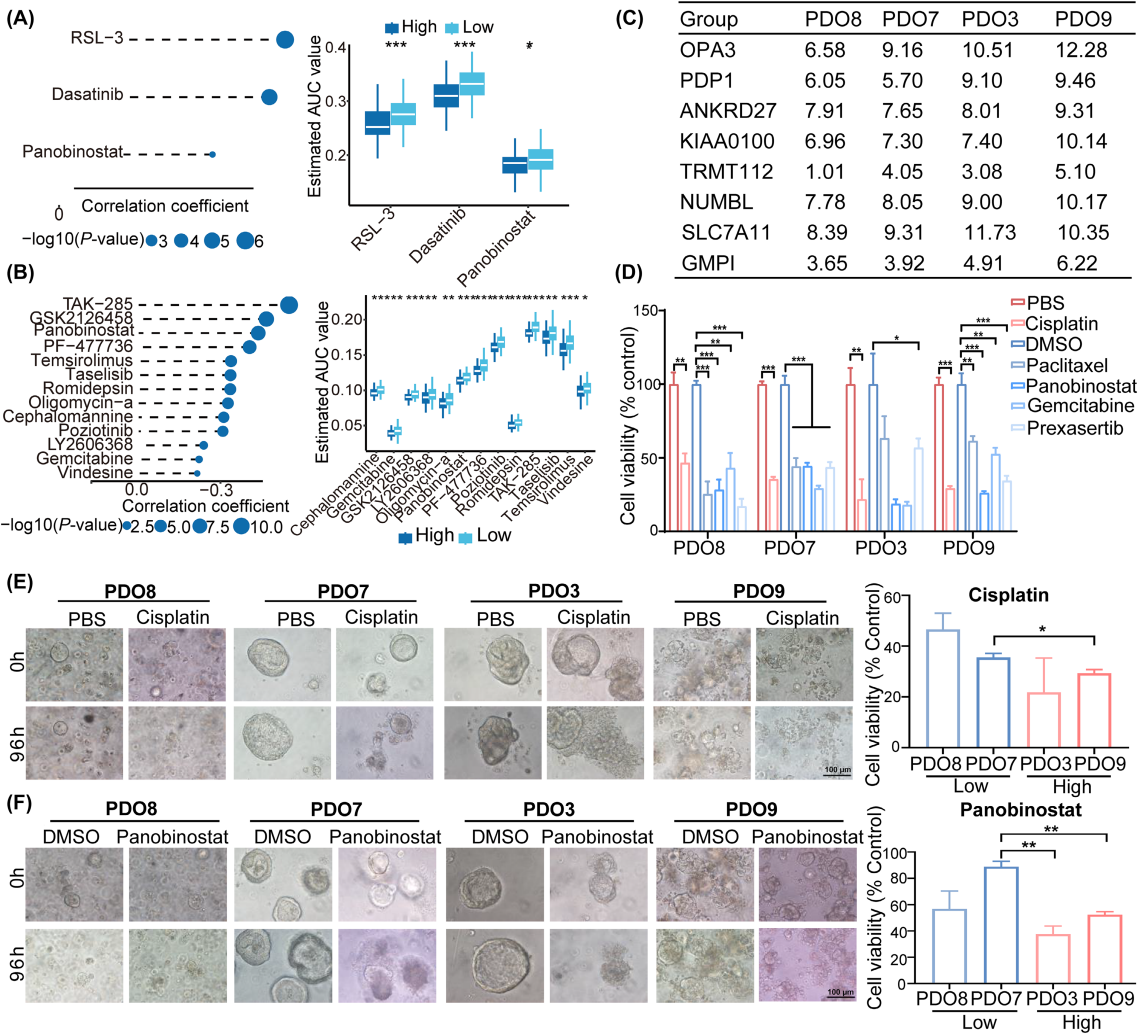
Evaluation of drug sensitivity based on PDOs with different GMPI. (A) Correlation between different AUC value of three potential drugs and GMPI based on CTRP database. (B) Correlation between different AUC values of 13 potential drugs and GMPI based on PRISM database. (C) GMPI index of each sample calculated based on mRNA expression levels of sevens genes in each sample. (D) Cell viability of PDOs treated with control, cisplatin, paclitaxel, gemcitabine, panobinostat and prexasertib. (E‐F) Representative bright‐field images and statistical histogram of organoids treated with cisplatin and panobinostat. Scale bar: 100 μm. *p < 0.05, **p < 0.01 and ***p < 0.001.

First, differential drug response analysis between low‐ and high‐GMPI groups was conducted to identify compounds with lower estimated AUC values in the high‐GMPI group. Next, spearman correlation analysis between AUC value and GMPI was used to identify compounds with negative correlation coefficients (Spearman's *r* < −0.05 for CTRP or −0.15 for PRISM). These results yielded three CTRP‐derived compounds (including RSL‐3, dasatinib and panobinostat; Figure 8A) and 13 PRISM‐derived compounds (including TAK‐285, GSK2126458, panobinostat, PF − 477,736, temsirolimus, taselisib, romidepsin, oligomycin‐a, cephalomannine, poziotinib, LY2606368, gemcitabine and vindesine) (Figure 8B). All these compounds displayed decreased estimated AUC values in high‐GMPI group and therapeutic efficiency of these compounds was negatively correlated with GMPI.

Patient‐derived organoids possess the characteristics of epithelial architecture and heterogeneity of the original tumour and have been proposed as alternative models for drug screening.^37^ To explore potential drugs targeting patients in the GMPI‐high group, we collected four ovarian cancer samples and calculated GMPI for each sample with the following formula: GMPI = 0.090* KIAA0100 + 0.043* ANKRD27 + 0.215* OPA3 + 0.171* NUMBL + 0.314* PDP1–0.233*SLC7A11–0.007* TRMT112. The PDOs generated from the four different samples were classified into high‐GMPI group (PDO3 and PDO9) and low‐GMPI group (PDO7 and PDO8) according to the median value of GMPI in the corresponding OC samples (Figure 8C). Subsequently, PDOs were treated with cisplatin, paclitaxel, gemcitabine, panobinostat and prexasertib at indicated concentration, respectively. The results showed that all the tested drug agents suppressed the growth of the PDOs from three OC samples (PDO7, PDO8 and PDO9) compared with those in control groups (all *p* < 0.05) (Figure 8D–F). PDO3 also exhibited decreased cell viability when treated with cisplatin or prexasertib (all *p* < 0.05), but exhibited modest effects after treatment with paclitaxel, panobinostat or gemcitabine (both *p* > 0.05; Figure 8D). Notably, PDOs with high GMPI showed greater cytotoxicity compared with those with low‐GMPI group when treated with cisplatin (PDO9 vs. PDO7) or panobinostat (PDO9 vs. PDO7 and PDO3 vs. PDO7; all *p* < 0.05; Figure 8E,F and Figure S9). However, PDOs with high GMPI displayed increased cell viability after treatment with paclitaxel (PDO9 vs. PDO8), gemcitabine (PDO9 vs. PDO7) or prexasertib (PDO3 vs. PDO8; Figure S10A,B). Collectively, these results suggest that GMPI could predict the therapeutic efficiency of drug candidates.

## 4 DISCUSSION

Glutamine metabolism is a fundamental and indispensable process involved in the development of tumour cells and immune cells in TME, providing promising strategies to inhibit tumour progression.^16^ Here, we first proposed a classification method to divide OC patients into three subtypes based on 20 GMRGs associated with prognosis. Patients in the three subtypes exhibited significantly different prognostic features, functional pathways, immune cell infiltration and immune functional status. These differential characteristics suggested that OC classification based on GM should be taken into consideration when developing personalised therapy. We further constructed a novel risk model GMPI to divide OC patients into low‐ and high‐GMPI groups based on the training (TCGA‐OV cohort) and external validation dataset (GEO cohort‐1). As expected, patients in high‐GMPI group presented a worse prognosis and poorer response to immunotherapy. The GMPI, which severed as an independent predictor for prognosis of OC, exhibited enhanced accuracy and potential implications in clinical practice, and its predictive performance was superior to that of all other assessed models. Overall, GMPI is characterized as a reliable model for predicting the prognosis and immunotherapeutic response in OC patients and may provide valuable insights into therapeutic strategies for OC.

All genes involved in GMPI model were associated with tumour progression, prognosis and immune regulation. KIAA0100 was an anti‐apoptotic factor associated with tumour carcinogenesis and progression, including acute monocytic leukaemia and breast cancer.^38^, ^39^ Dominant‐negative mutations of NUMBL and NUMB resulted in Treg cells dysfunction through activating Notch1/CD22 signalling axis.^40^ Pyruvate dehydrogenase phosphatase 1 (PDP1) could accelerate intracellular ATP production and promote cancer progression through mTOR activation.^41^ SLC7A11 deletion in macrophage led to the recruitment of CD8^+^ T cells and the activation of IFN‐γ‐induced JAK/STAT1 axis.^42^ TRMT112 not only participated in multiple biological processes, including cell proliferation and DNA damage,^43^ but also exhibited positive correlation with various immune cells, such as dendritic cells, T cells and macrophages.^44^ In summary, GMPI is likely to be a prognostic indicator to evaluate the aggressiveness and immune status of OC.

Patients in low‐ and high‐GMPI groups exhibited differences in survival time, cancer functions, immune infiltration and TME landscape. Notably, in the present study, a variety of well‐recognised oncogenic signalling pathways, such as oxidative phosphorylation, TGF‐beta, PI3K‐AKT, Ras, P53 and HIF‐1α pathways, were increased in the high‐GMPI group. Glutamine deprivation might promote the migration and invasion of colon cancer cells by inducing the EMT process.^45^ P53 promoted glutamine metabolism in tumour cells through upregulating GLS2.^46^ HIF‐1α inhibition reduced glutamine consumption in tumour cells.^47^ Accordingly, tumour characterised by high‐GMPI can be regarded as a representative of high malignancy, which provides additional insights into the function mechanisms of GMPI in OC, and combined therapies targeting GM and oncogenic pathways are expected to be a promising treatment strategy for OC.

It's reported that GM impacted the distribution and function of immune cells in TME. Loss of GLS could promote the differentiation and effector function of CD8 T cells.^48^ Glutamine deprivation or withdrawal promoted Treg cells differentiation to induce immunosuppression^49^ or inhibited NK cell proliferation and antitumor functions.^50^ Glutamine not only boosted M2 macrophages polarisation by facilitating the biosynthesis of M2‐related proteins, such as CD206, KLF4, CCL22 and IRF4,^51^ but also enhanced the formation of superoxide in neutrophils.^52^ Our research showed that high‐GMPI group harboured high levels of DCs, macrophages, MDSCs, regulatory T cells, neutrophils and exhibited a high frequency of various immune functions, such as APC co‐inhibition, APC co‐stimulation, type II IFN response. The unfavourable survival outcome of patients in the high‐GMPI group can be attributed to the increased infiltration of immunosuppressive cells, which can impede anti‐tumour CD8^+^ T cells infiltration, resulting in immune dysfunction and an increased risk of immune escape. Interestingly, single‐cell analysis revealed that OC cells in low‐ and high‐GMPI groups could communicate with immunocytes through multiple potential ligand‐receptor pairs, including GRN‐SORT1,^53^ MDK‐SDC4^54^ and TNFSF12‐TNFRSF12A,^55^ indicating that GM can serve as a bridge between OC cells and immunocytes infiltration to affect the progression and treatment of OC.

Glutamine starvation could increase the expression of PD‐L1 on tumour cells.^56^ The glutaminase antagonist JHU‐083 was demonstrated to enhance oxidative phosphorylation and bolster anticancer immune responses of CD8 T cells.^14^ We found that patients in low‐GMPI group may have a better response to immunotherapies, and GMPI served as a potential predictor of immunotherapy response in OC patients. PDOs, which recapitulated tumour histological features, have been emerged as a reliable preclinical model for exploring therapeutic response and guiding therapeutic decisions.^37^ In this work, we analysed the sensitivity of various drug candidates in different GMPI groups with PDOs. These drugs assessed included cisplatin, paclitaxel, gemcitabine, panobinostat and prexasertib. We observed pronounced inhibitory effects of these drugs on the four PDOs. Specifically, cisplatin and panobinostat exhibited greater suppression rates in high‐GMPI group, while paclitaxel and prexasertib presented increased cytotoxicity in low‐GMPI group. Our findings suggest that cisplatin and panobinostat are more effective in treating OC patients with high GMPI, and paclitaxel and prexasertib show greater effectiveness in OC patients with low GMPI. These findings underscore the potential of GMPI as a valuable tool for making appropriate therapeutic options and facilitating clinical translation for OC patients.

The study has several advantages. Firstly, we identified three molecular clusters of patients based on the prognostic GMRGs, revealing different prognosis and immune statuses within these clusters. Secondly, the construction of GMPI for each individual demonstrated that patients with a lower GMPI presented a favourable prognosis, and tumour cells with different GMPI could communicate with distinct cell populations at single‐cell level. Thirdly, we validated the therapeutic efficiencies of several drug candidates using PDOs with different GMPI, thereby facilitating their clinical application under the guidance of GMPI. Although our work yielded promising results, several limitations need to be clarified. Firstly, a large‐scale, multi‐center prospective study is necessary to further validate the efficacy of the GMPI model. Secondly, in vitro and in vivo experimental validations are essential to elucidate the biological functions of GMPI‐related genes in OC. Lastly, although we predicted the therapeutic efficacy of several drugs in different GMPI groups, it is necessary to validate these predictions through in vivo drug assays and clinical trials.

In summary, we constructed a metabolism‐related GMPI model with the aim to predict the prognosis and therapeutic efficacy of OC patients. We elucidated the differences in patient characteristics based on the prognostic values, functional pathways, immune infiltration and therapeutic regimens between the different GMPI groups. These preliminary findings strongly implied that GMPI can be identified as prognostic classifiers for OC, potentially guiding innovative perspectives on effective therapeutic strategies and contributing to a deeper understanding of the molecular mechanisms of OC. Specially, PDO models provided fundamental validation of GMPI's role in drug screening and therapeutic efficacy. The application of GMPI will expedite prognosis prediction and the development of treatment strategies for OC patients.

## AUTHOR CONTRIBUTIONS

**Lingling Gao:** Conceptualization (lead); funding acquisition (equal); investigation (lead); writing – original draft (lead). **Zheng Wei:** Data curation (equal); investigation (equal); methodology (equal); validation (equal). **Feiquan Ying:** Formal analysis (equal); methodology (equal); software (equal). **Lin Huang:** Formal analysis (equal); software (equal). **Jingni Zhang:** Data curation (equal); formal analysis (equal). **Si Sun:** Visualization (equal); writing – review and editing (equal). **Zehua Wang:** Conceptualization (equal); supervision (equal). **Jing Cai:** Conceptualization (equal); supervision (equal); writing – review and editing (equal). **Yuan Zhang:** Funding acquisition (equal); supervision (equal); writing – review and editing (equal).

## FUNDING INFORMATION

This work was supported by National Natural Science Foundation of China (grant number: 82203803) and Key Research and Development Program of Hubei Province (grant numbers: 2021BCA118).

## CONFLICT OF INTEREST STATEMENT

The authors declare that there are no conflicts of interest.

## Supporting information


Appendix S1.

## Data Availability

All data generated or analysed during this study are collected from the Cancer Genome Atlas (TCGA, https://portal. gdc.cancer.gov/) and Gene Expression Omnibus (GEO, https://www. ncbi.nlm.nih.gov/geo/).
