# Supplementary material for: Glutamine metabolism prognostic index predicts tumour microenvironment characteristics and therapeutic efficacy in ovarian cancer
Source: J Cell Mol Med. 2024 Mar 20;28(7):e18198. doi: 10.1111/jcmm.18198 (PMC10951877; doi:10.1111/jcmm.18198)
Supplement: Supplementary file 1 — Appendix S1. [file JCMM-28-e18198-s001.docx]

**Supplementary materials**


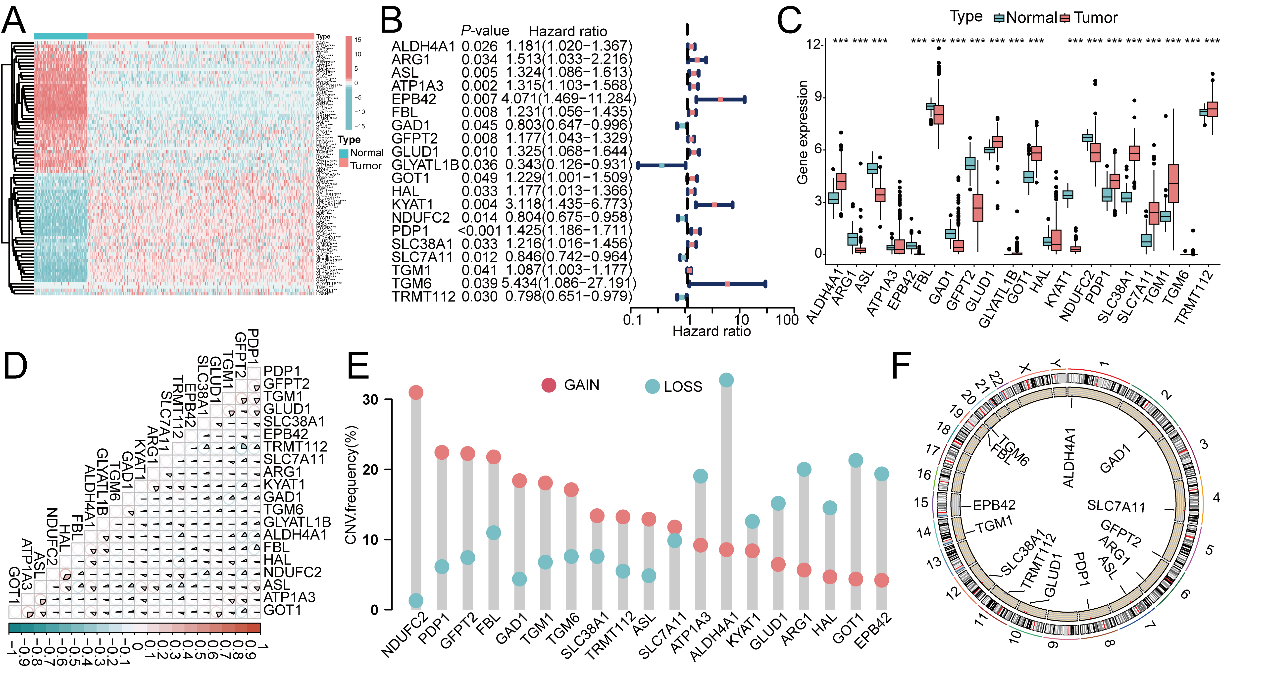


**Supplementary Figure 1 Genetic and transcriptional alterations of GMRGs in OC.** **(A)** The expression profile of seventy-seven of one hundred and thirty-five GMRGs in normal and ovarian cancer tissues. **(B)** Identification of twenty GMRGs associated with prognosis using univariate Cox regression analysis. **(C)** Differential expression of twenty GMRGs between normal and ovarian cancer tissues. **(D)** Correlation analysis among 20 GMRGs in OC. Blue represents negative correlation, red represents positive correlation. **(E)** CNV mutation frequency of twenty GMRGs in OC. Blue represents deletion, red represents amplification. **(F)** The location of CNV alterations of GMRGs on chromosomes. **P* < 0.05; ***P* < 0.01; ****P* < 0.001).


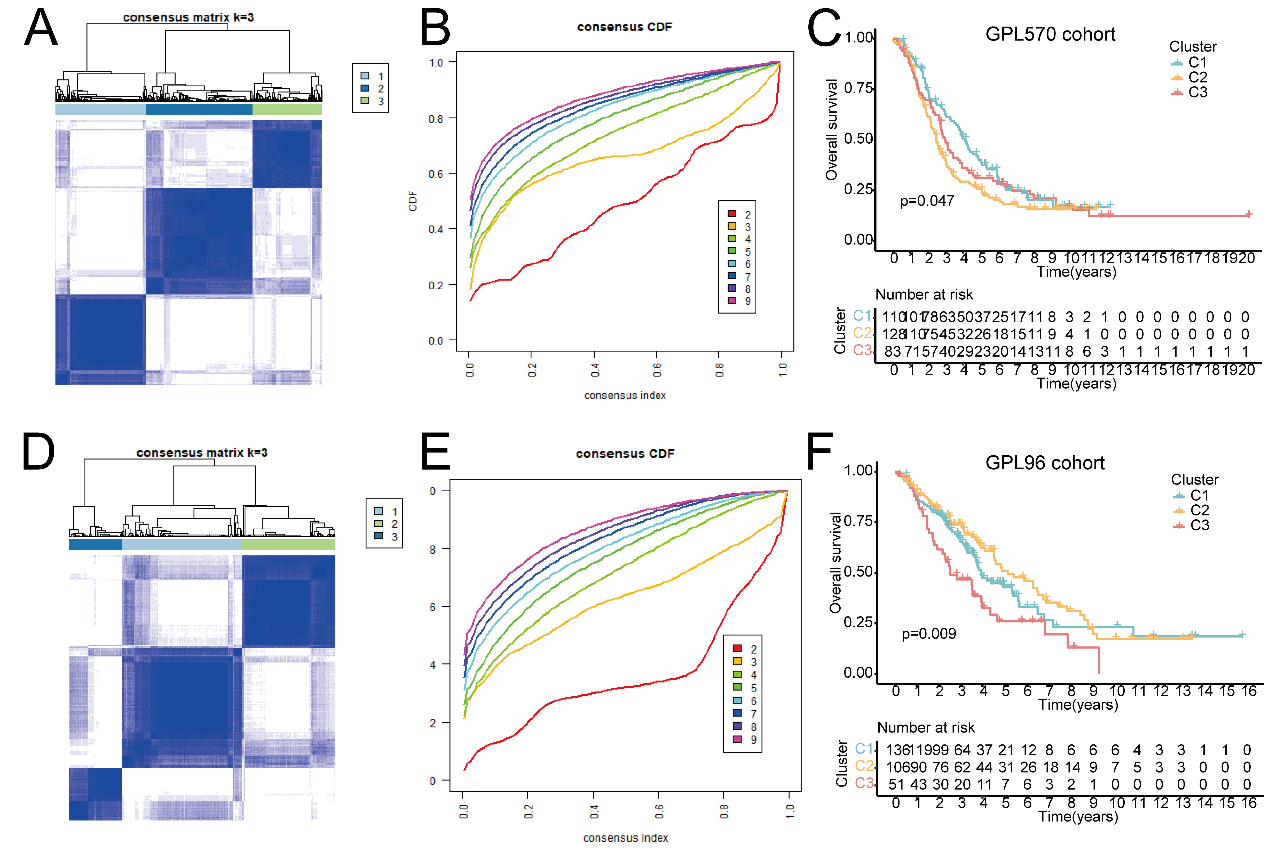


**Supplementary Figure 2 Identification of three clusters based on two cohorts. (A)** Consensus clustering matrix of sample under *k* = 3 based on GPL570 cohort. **(B)** Cumulative distribution curve with the number of subtypes *k* = 2 to 9 based on GPL570 cohort. **(C)** Kaplan-Meier curves of OS among three clusters in GPL570 cohort. **(D)** Consensus clustering matrix of sample under *k* = 3 based on GPL96 cohort. **(B)** Cumulative distribution curve with the number of subtypes *k* = 2 to 9 based on GPL96 cohort. **(F)** Kaplan-Meier curves of OS among three clusters in GPL96 cohort.


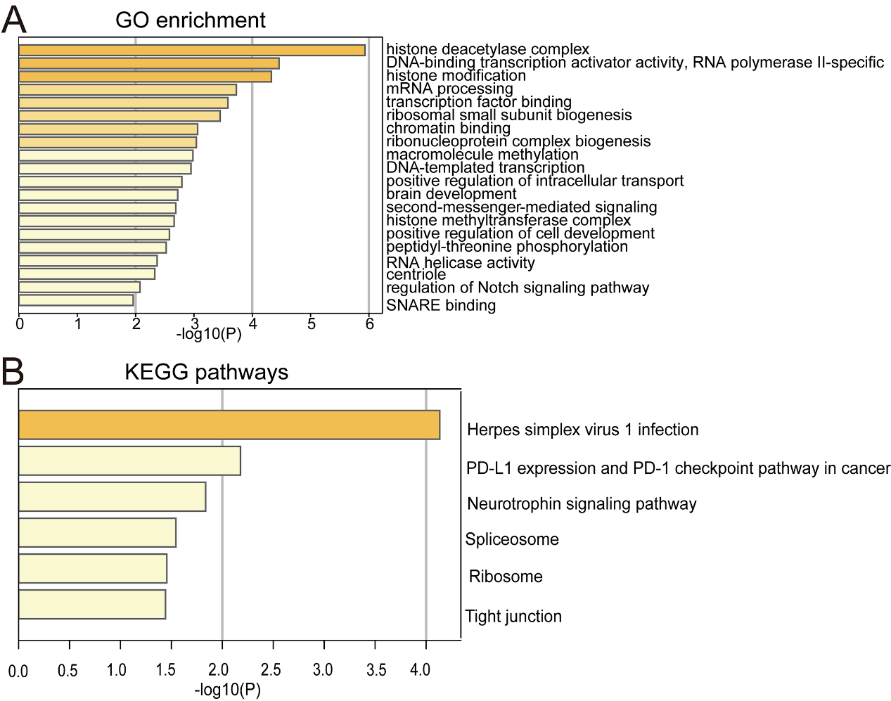


**Supplementary Figure 3 Functional enrichment of DEGs among three GM clusters. (A)** GO enrichment analysis of 143 DEGs among three GM clusters. **(B)** KEGG enrichment analysis of 143 DEGs among three GM clusters.


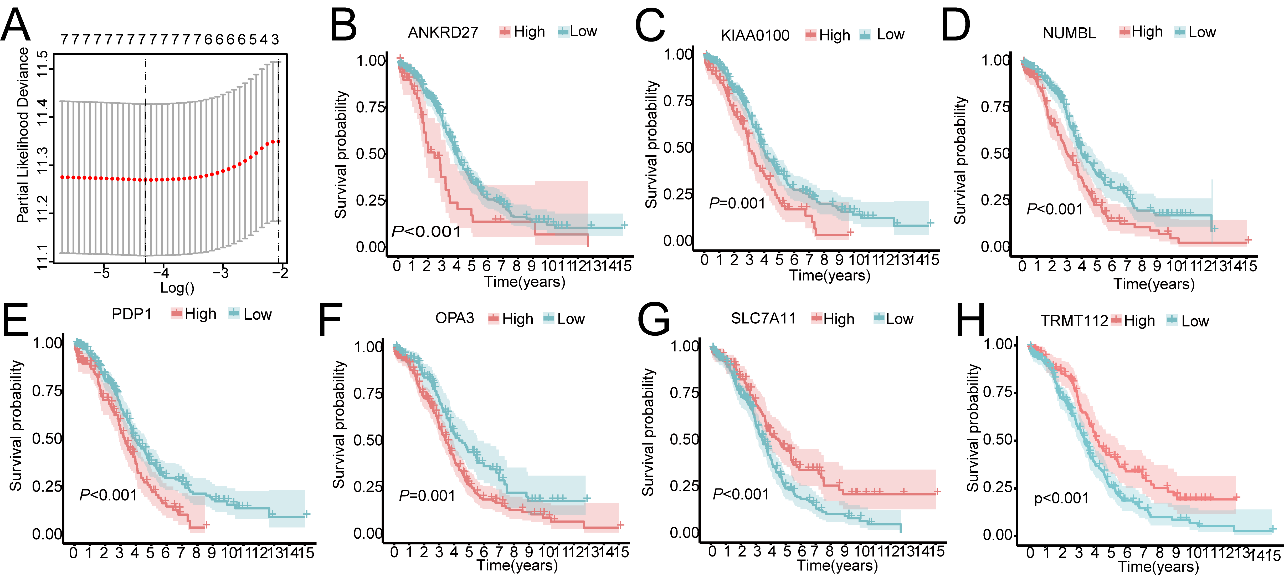


**Supplementary Figure 4 Correlation analysis of GMPI in OC. (A)** Validation of the LASSO Cox regression model for the tuning parameter selection. The horizontal axis represents the log (lambda) value, and the vertical axis represents partial likelihood deviance. The red dots represent partial likelihood deviance for a tuning parameter, and error bars represent standard errors. **(B-H)** Prognostic evaluation of ANKRD27 **(B)**, KIAA0100 **(C)**, NUMBL **(D)**, PDP1 **(E)**, OPA3 **(F)**, SLC7A11 **(G)** and TRMT112 **(H)** in GMPI model by KM curves.


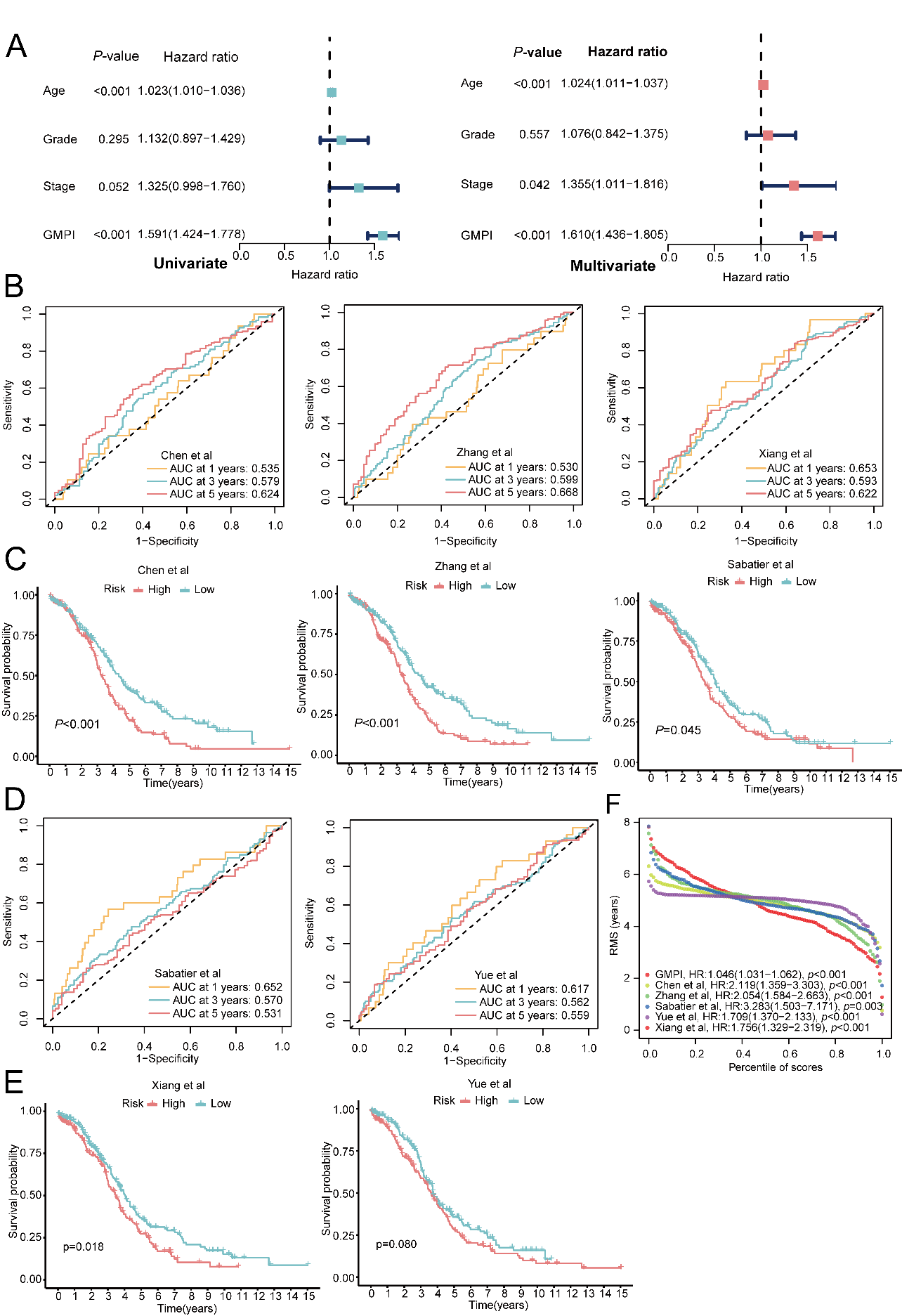


**Supplementary Figure 5 Comparison of GMPI model with other established signatures. (A**) The relationship between GMPI, the clinical characteristics and OS in OC with univariate and multivariate Cox analysis. **(B-E)** ROC and KM curves of other five published signatures. **(F)** Restricted mean survival (RMS) time curve of all six prognostic risk models.


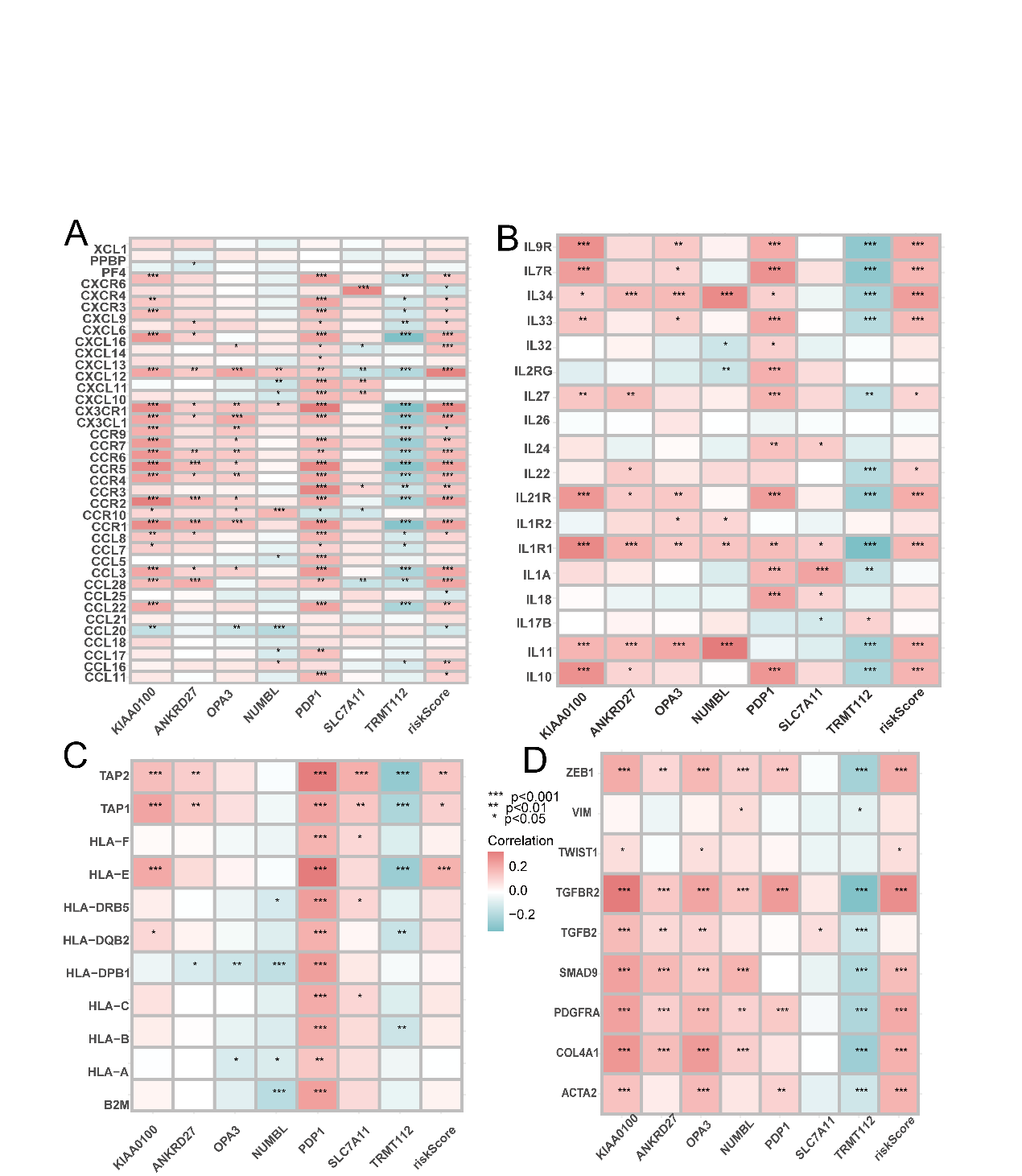


**Supplementary Figure 6** **Correlation between GMPI and various molecules. (A-D)** Correlation analysis of chemokines **(A)**, interleukins **(B)**, MHC **(C)** and regulators related to TGF-β/EMT pathway **(D)** with GMPI and seven genes.


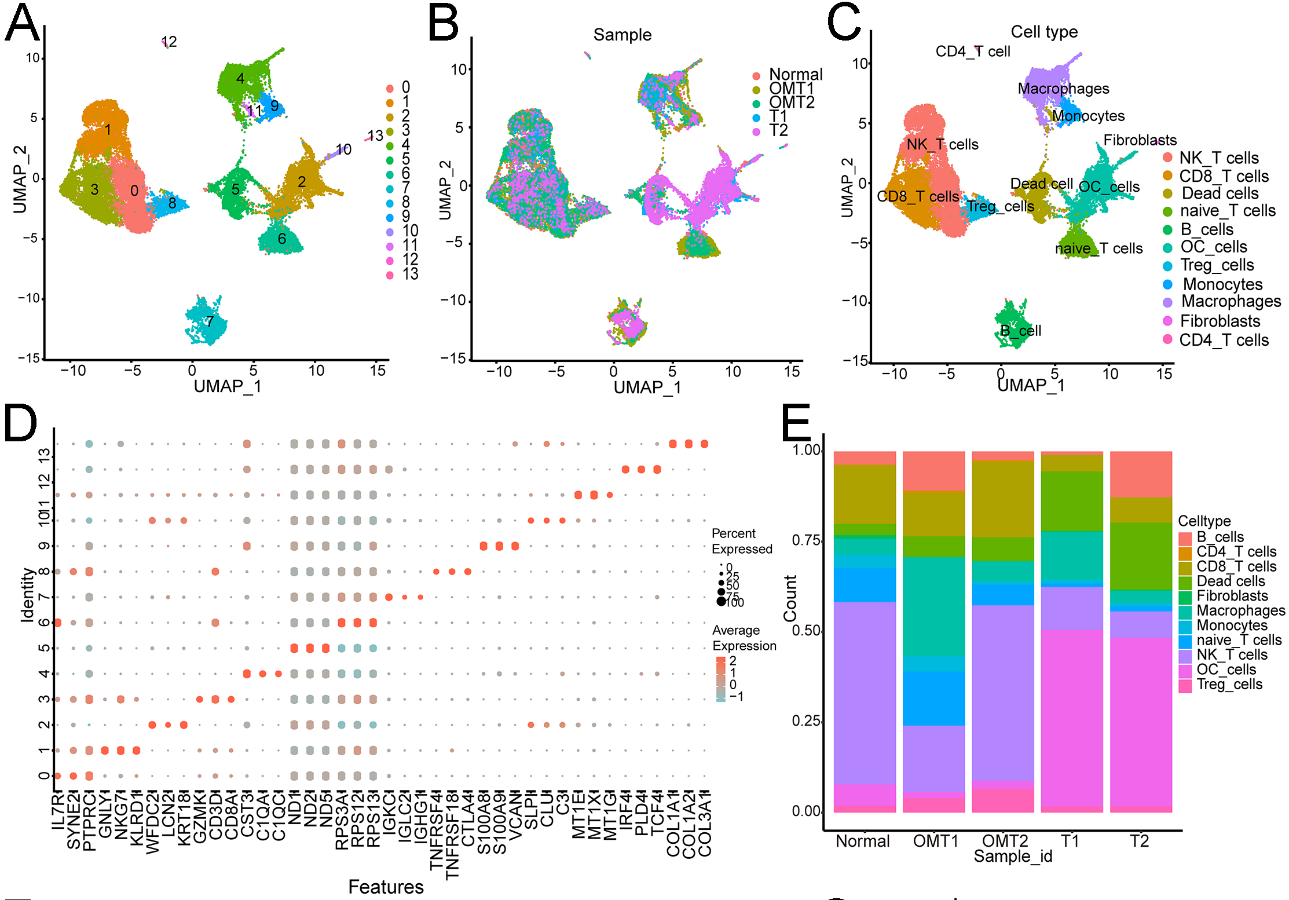


**Supplementary Figure 7 Identified fourteen clusters after cellular quality based on scRNA-seq dataset (GSE181955). (A)** Fourteen cell clusters shown by UMAP plot. **(B)** The sample origin of the cells shown by UMAP. **(C)** Distinct cell types identified by marker genes with UMAP plot. **(D)** The expression levels of specific marker genes in each cell type shown by bubble plots. **(E)** Proportion of each cell type in each cell type shown by bar plots.


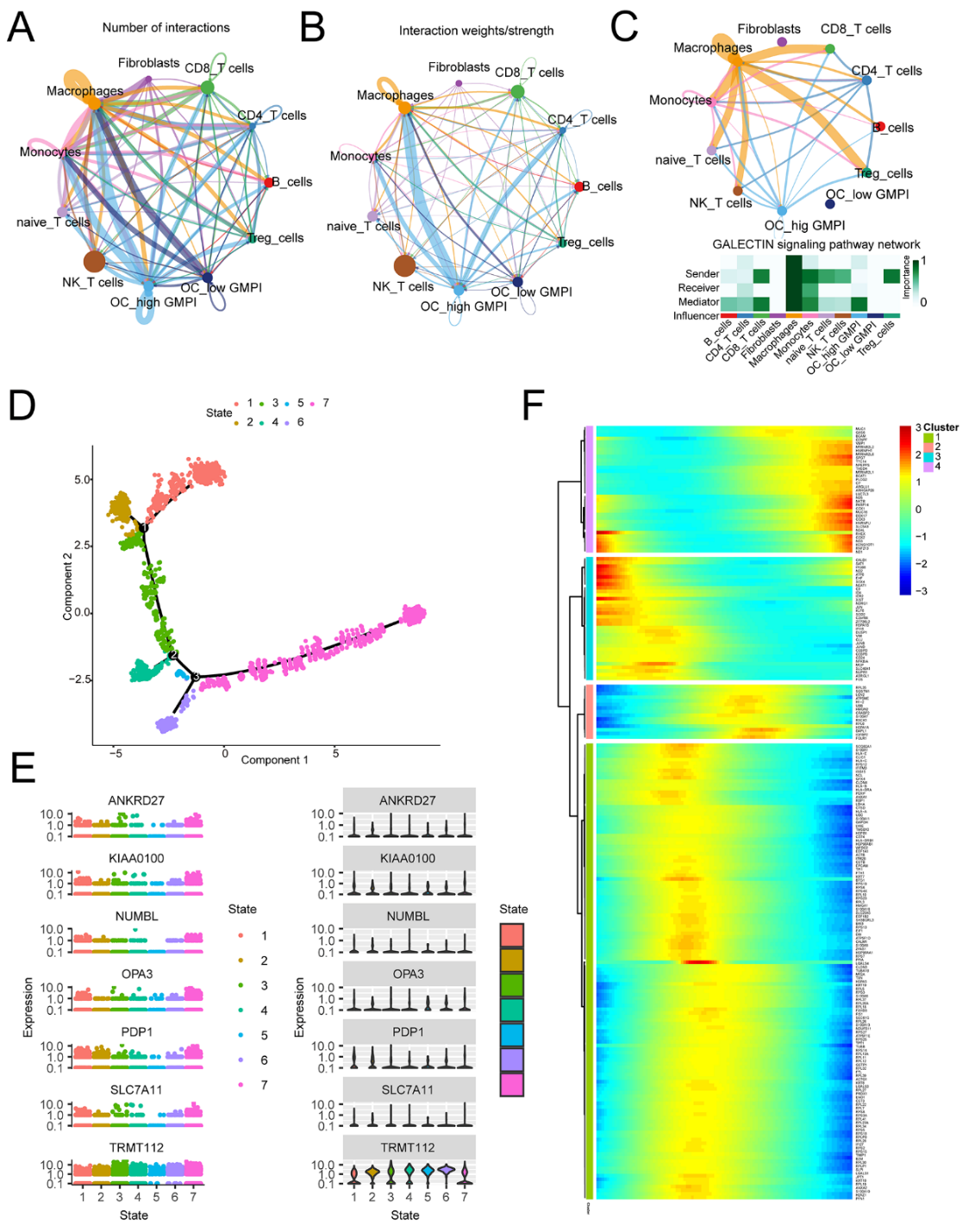


**Supplementary Figure 8 Intercellular communication and evolutionary trajectory of different GMPI groups in OC**. **(A-B)** Number of interactions and interactions weight/length between various cell types and malignant cells in low- and high- GMPI groups with circle plot. **(C)** High GMPI cells communicate with various cell types through GALECTIN signaling pathways with circle plots. **(D)** Pseudotime analysis of trajectory differentiation of OC cells by cell state. **(E)** Relative expression of GMPI-related seven genes in the differentiation process of OC cells colored by cell states. **(F)** Dynamic expression under trajectory differentiation of OC cells with heatmap.


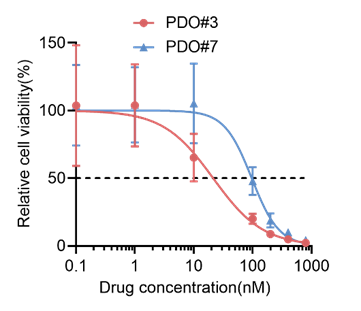


**Supplementary Figure 9** The cell viability of PDO3 and PDO7 treated with panobinostat.


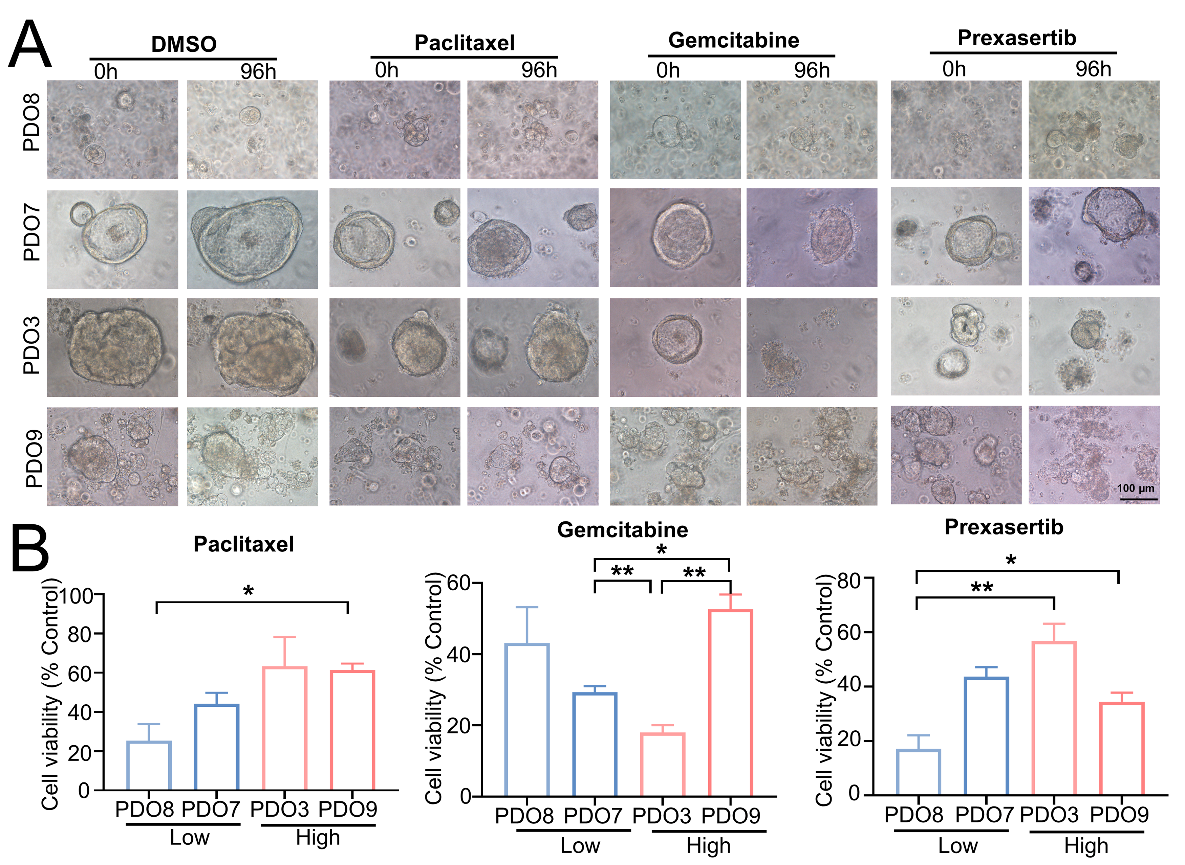


**Supplementary Figure 10 Drug sensitivity of PDOs. (A)** Representative bright-field images of organoids treated with paclitaxel, gemcitabine and prexasertib. Scale bar: 100 μm. **(B)** Statistical histogram of cell viability of PDOs treated with paclitaxel, gemcitabine and prexasertib. **P* < 0.05, ***P* < 0.01, and ****P* < 0.001.

**TableS1 Basic characteristics of datasets enrolled in this study**

| **Datasets** | **Platform** | **Numbers** | **Survival** |
| --- | --- | --- | --- |
| TCGA-OV | TCGA | 562 | OS |
| GSE14764 | GPL96 | 80 | OS |
| GSE23554 | GPL96 | 28 | OS |
| GSE26712 | GPL96 | 185 | OS |
| GSE26193 | GPL570 | 107 | OS |
| GSE63885 | GPL570 | 75 | OS |
| GSE18520 | GPL570 | 53 | OS |
| GSE19829 | GPL570 | 28 | OS |
| GSE30161 | GPL570 | 58 | OS |
| GSE32062 | GPL6480 | 260 | OS |
| GSE17260 | GPL6480 | 110 | OS |
| GSE140082 | GPL14951 | 380 | OS |
| GSE49997 | GPL2986 | 194 | OS |

**Table S2 The sequences of primers used in qRT-PCR.**

| **Gene** | **Primer** | **Sequence (5'-3')** |
| --- | --- | --- |
| *ANKRD27* | Forward | TTGACAGGAACATCGCCTCTT |
|  | Reverse | GAGAGCATTCGCTGAGTCTATG |
| *OPA3* | Forward | CCTATGGCGAAGCTGCTATAC |
|  | Reverse | GGCGGCCTCCTTAATACGG |
| *NUMBL* | Forward | TGGTGGACGACAAAACCAAGG |
|  | Reverse | ACGACAGATATAGGAGAAAGCCT |
| *PDP1* | Forward | TGTTCCTCATCGTACATTCCTCA |
|  | Reverse | AGAACTGACATTTTTGCCGTCAA |
| *SLC7A11* | Forward | TCTCCAAAGGAGGTTACCTGC |
|  | Reverse | AGACTCCCCTCAGTAAAGTGAC |
| *TRMT112* | Forward | ATAACTTGCGTCTGATCCAGGT |
|  | Reverse | GGTGCCCTCTATCACTTCCAC |
| *KIAA0100* | Forward | TGCTGCTAGTTGCGCTTAG |
|  | Reverse | TTCCGCTGACACCACTTGG |
| *GAPDH* | Forward | GTCATGGGTGTGAACCATGAGAAG |
|  | Reverse | AGTCTTCTGGGTGGCAGTGATG |

**Table S3 Prognostic values of twenty GMRGs in OC patients from TCGA-OV dataset**

| **id** | **HR** | **HR.95L** | **HR.95H** | ***P*-value** |
| --- | --- | --- | --- | --- |
| ALDH4A1 | 1.180671 | 1.019789 | 1.366934 | 0.02627268 |
| ARG1 | 1.512579 | 1.032556 | 2.215757 | 0.03363352 |
| ASL | 1.323794 | 1.086493 | 1.612924 | 0.00538588 |
| ATP1A3 | 1.314933 | 1.102812 | 1.567853 | 0.00228635 |
| EPB42 | 4.071364 | 1.468989 | 11.28396 | 0.00694712 |
| FBL | 1.231441 | 1.056479 | 1.435379 | 0.0077526 |
| GAD1 | 0.802557 | 0.646987 | 0.995533 | 0.0454266 |
| GFPT2 | 1.177324 | 1.042912 | 1.329058 | 0.00830827 |
| GLUD1 | 1.32524 | 1.068329 | 1.643932 | 0.01043387 |
| GLYATL1B | 0.342699 | 0.126139 | 0.931059 | 0.03572494 |
| GOT1 | 1.229088 | 1.001196 | 1.508851 | 0.04867906 |
| HAL | 1.176677 | 1.013351 | 1.366327 | 0.03284976 |
| KYAT1 | 3.117841 | 1.435289 | 6.772805 | 0.0040667 |
| NDUFC2 | 0.804118 | 0.675179 | 0.957682 | 0.01449009 |
| PDP1 | 1.424776 | 1.186116 | 1.711458 | 0.00015388 |
| SLC38A1 | 1.216203 | 1.016222 | 1.455537 | 0.03271713 |
| SLC7A11 | 0.845896 | 0.742133 | 0.964168 | 0.01219477 |
| TGM1 | 1.08684 | 1.003249 | 1.177397 | 0.04141065 |
| TGM6 | 5.434244 | 1.086056 | 27.19105 | 0.03935545 |
| TRMT112 | 0.798318 | 0.651093 | 0.978833 | 0.03033742 |

**Table S4 Prognostic analysis of 49 DEGs using univariate Cox analysis.**

| **id** | **HR** | **HR.95L** | **HR.95H** | ***P*-value** |
| --- | --- | --- | --- | --- |
| POLR1A | 1.017129 | 1.00312 | 1.031333 | 0.016383 |
| KMT2B | 1.011767 | 1.004655 | 1.01893 | 0.001153 |
| PLEKHM3 | 1.070836 | 1.017873 | 1.126555 | 0.008182 |
| GCN1 | 1.005589 | 1.000753 | 1.010448 | 0.023437 |
| MEGF8 | 1.010602 | 1.003577 | 1.017676 | 0.003044 |
| ZNF142 | 1.033178 | 1.007514 | 1.059495 | 0.010981 |
| PPRC1 | 1.008103 | 1.000865 | 1.015393 | 0.028154 |
| RPS16 | 1.000081 | 1.000003 | 1.000159 | 0.041988 |
| NFKBID | 1.011331 | 1.000514 | 1.022266 | 0.040019 |
| KIAA0100 | 1.008785 | 1.005379 | 1.012202 | 3.99E-07 |
| SIPA1L3 | 1.005856 | 1.000777 | 1.010961 | 2.38E-02 |
| LRFN3 | 1.014169 | 1.00231 | 1.026169 | 0.01906 |
| PHF12 | 1.015986 | 1.006994 | 1.025058 | 0.000471 |
| UTP20 | 1.040738 | 1.006693 | 1.075934 | 0.018618 |
| WIZ | 1.00704 | 1.002108 | 1.011995 | 0.005098 |
| ANKRD27 | 1.013031 | 1.004834 | 1.021295 | 0.001789 |
| HIF1AN | 1.031723 | 1.006204 | 1.057889 | 0.014526 |
| AC106820.1 | 1.047445 | 1.011645 | 1.084511 | 0.008988 |
| SLC35E1 | 1.006373 | 1.002176 | 1.010588 | 0.002888 |
| GPATCH1 | 1.021378 | 1.000671 | 1.042512 | 0.042951 |
| AKAP8L | 1.009947 | 1.002086 | 1.017869 | 0.01304 |
| AC073046.1 | 1.024198 | 1.004648 | 1.044128 | 0.015033 |
| RBM19 | 1.020322 | 1.000933 | 1.040086 | 0.039853 |
| CDK19 | 1.018101 | 1.000315 | 1.036205 | 0.046048 |
| ZNF146 | 1.003759 | 1.001234 | 1.006291 | 0.003502 |
| ZNF507 | 1.033566 | 1.006529 | 1.061328 | 0.014639 |
| CIC | 1.00292 | 1.000516 | 1.00533 | 0.017262 |
| MRPS12 | 1.001495 | 1.000356 | 1.002635 | 0.010099 |
| ZNF8 | 1.040001 | 1.014209 | 1.066449 | 0.002205 |
| OPA3 | 1.057295 | 1.029603 | 1.085731 | 3.88E-05 |
| ZNF526 | 1.023275 | 1.00443 | 1.042473 | 1.53E-02 |
| RPL23AP65 | 1.018273 | 1.007283 | 1.029383 | 1.07E-03 |
| PLCG1 | 1.009455 | 1.000443 | 1.018549 | 3.97E-02 |
| SYMPK | 1.010857 | 1.003071 | 1.018703 | 6.20E-03 |
| AC011465.1 | 1.041271 | 1.000816 | 1.083362 | 4.55E-02 |
| KDM2B | 1.024315 | 1.000156 | 1.049057 | 4.85E-02 |
| ARHGEF17 | 1.004396 | 1.000536 | 1.00827 | 2.56E-02 |
| FAM222B | 1.011703 | 1.004854 | 1.0186 | 7.88E-04 |
| ZNF384 | 1.009942 | 1.002143 | 1.0178 | 1.24E-02 |
| PROSER3 | 1.014028 | 1.000221 | 1.028025 | 4.64E-02 |
| NUMBL | 1.005885 | 1.001394 | 1.010397 | 1.02E-02 |
| CHERP | 1.004577 | 1.001337 | 1.007827 | 5.59E-03 |
| DMPK | 1.019922 | 1.006796 | 1.03322 | 2.84E-03 |
| AC115223.1 | 1.010446 | 1.002223 | 1.018736 | 1.27E-02 |
| CAPN10 | 1.036 | 1.001591 | 1.071591 | 4.01E-02 |
| GGCX | 1.031776 | 1.01289 | 1.051015 | 9.04E-04 |
| SIX5 | 1.016326 | 1.003549 | 1.029266 | 1.21E-02 |
| FBXL14 | 1.006226 | 1.000601 | 1.011883 | 3.00E-02 |
| STK11IP | 1.026203 | 1.004991 | 1.047862 | 1.52E-02 |

**Table S5 Correlation between GMPI and tumor-infiltrating immune cells evaluated by various software**

| **Immune cell** | **cor** | ***P*-value** |
| --- | --- | --- |
| T cell CD4+_TIMER | 0.182806217 | 0.002765243 |
| T cell CD8+_TIMER | 0.144404374 | 0.018451223 |
| Neutrophil_TIMER | 0.272484756 | 6.52E-06 |
| Macrophage_TIMER | 0.408049201 | 4.28E-12 |
| Myeloid dendritic cell_TIMER | 0.225681052 | 0.000206111 |
| B cell naive_CIBERSORT | 0.129898787 | 0.034209991 |
| B cell plasma_CIBERSORT | -0.134321778 | 0.028499576 |
| B cell naive_CIBERSORT-ABS | 0.178706748 | 0.003450429 |
| T cell CD4+ memory resting_CIBERSORT-ABS | 0.196976657 | 0.001241556 |
| T cell regulatory (Tregs)_CIBERSORT-ABS | 0.127525617 | 0.037656083 |
| Macrophage M2_CIBERSORT-ABS | 0.226761168 | 0.000191749 |
| Mast cell resting_CIBERSORT-ABS | 0.122279441 | 0.046326305 |
| Macrophage M1_QUANTISEQ | 0.227731227 | 0.000179654 |
| Macrophage M2_QUANTISEQ | 0.175702739 | 0.004046359 |
| Neutrophil_QUANTISEQ | 0.133760307 | 0.029175939 |
| NK cell_QUANTISEQ | 0.251013253 | 3.46E-05 |
| T cell regulatory (Tregs)_QUANTISEQ | 0.16452672 | 0.007165318 |
| uncharacterized cell_QUANTISEQ | -0.22318488 | 0.000243232 |
| T cell_MCPCOUNTER | 0.149256743 | 0.014830047 |
| NK cell_MCPCOUNTER | 0.215658108 | 0.000396371 |
| Monocyte_MCPCOUNTER | 0.297336811 | 7.86E-07 |
| Macrophage/Monocyte_MCPCOUNTER | 0.297336811 | 7.86E-07 |
| Neutrophil_MCPCOUNTER | 0.401036023 | 1.07E-11 |
| Endothelial cell_MCPCOUNTER | 0.310184924 | 2.43E-07 |
| Cancer associated fibroblast_MCPCOUNTER | 0.284966254 | 2.31E-06 |
| T cell CD4+ memory_XCELL | -0.143658174 | 0.019071445 |
| T cell CD4+ naive_XCELL | 0.13431979 | 0.028501946 |
| T cell CD8+ naive_XCELL | -0.159066944 | 0.009359066 |
| Common lymphoid progenitor_XCELL | -0.23496991 | 0.000109509 |
| Granulocyte-monocyte progenitor_XCELL | 0.158490077 | 0.009622535 |
| Hematopoietic stem cell_XCELL | 0.141657193 | 0.020824849 |
| Macrophage M1_XCELL | 0.157424957 | 0.010126324 |
| T cell NK_XCELL | 0.127514293 | 0.037673206 |
| B cell plasma_XCELL | -0.147457529 | 0.016092767 |
| T cell CD4+ Th2_XCELL | -0.132511753 | 0.03072936 |
| T cell regulatory (Tregs)_XCELL | 0.12910471 | 0.035331968 |
| Cancer associated fibroblast_EPIC | 0.299339487 | 6.57E-07 |
| Endothelial cell_EPIC | 0.253597373 | 2.85E-05 |
| Macrophage_EPIC | 0.216923728 | 0.000365545 |
| uncharacterized cell_EPIC | -0.354239332 | 2.78E-09 |
